# Cutting-edge developments in zinc oxide nanoparticles: synthesis and applications for enhanced antimicrobial and UV protection in healthcare solutions

**DOI:** 10.1039/d4ra02452d

**Published:** 2024-07-03

**Authors:** Egwonor Loveth Irede, Raymond Femi Awoyemi, Babatunde Owolabi, Omowunmi Rebecca Aworinde, Rofiat Odunayo Kajola, Ajibola Hazeez, Ayuba Adawale Raji, Latifat Oluwatobi Ganiyu, Chimezie O. Onukwuli, Asishana Paul Onivefu, Ikhazuagbe Hilary Ifijen

**Affiliations:** a Department of Chemistry, Clemson University Clemson South Carolina USA; b Department of Chemistry, Mississippi State University Starkville Mississippi MS 39762 USA; c Department of Civil Engineering, University of Alabama Tuscaloosa Alabama AL 35487 USA; d Department of Chemistry, Michigan Technological University Houghton Michigan USA; e Department of Biomedical Engineering, University of Rochester 500 Joseph C. Wilson Blvd. Rochester NY 14627 USA; f Department of Urban and Regional Planning, University of Lagos Lagos Nigeria; g Department of Surveying and Geo-Informatics, Bells University of Technology Ota Ogun State Nigeria; h Department of Chemistry, Brigham Young University Provo Utah USA; i Department of Chemistry, Eastern New Mexico University Portales New Mexico USA; j Department of Chemistry and Biochemistry, University of Delaware Newark DE 19716 USA; k Department of Research Outreach, Rubber Research Institute of Nigeria Iyanomo Benin City Nigeria larylans4u@yahoo.com ifijen.hilary@rrin.gov.ng

## Abstract

This paper presents a comprehensive review of recent advancements in utilizing zinc oxide nanoparticles (ZnO NPs) to enhance antimicrobial and UV protective properties in healthcare solutions. It delves into the synthesis techniques of ZnO NPs and elucidates their antimicrobial efficacy, exploring the underlying mechanisms governing their action against a spectrum of pathogens. Factors impacting the antimicrobial performance of ZnO NPs, including size, surface characteristics, and environmental variables, are extensively analyzed. Moreover, recent studies showcasing the effectiveness of ZnO NPs against diverse pathogens are critically examined, underscoring their potential utility in combatting microbial infections. The study further investigates the UV protective capabilities of ZnO NPs, elucidating the mechanisms by which they offer UV protection and reviewing recent innovations in leveraging them for UV-blocking applications in healthcare. It also dissects the factors influencing the UV shielding performance of ZnO NPs, such as particle size, dispersion quality, and surface coatings. Additionally, the paper addresses challenges associated with integrating ZnO NPs into healthcare products and presents future perspectives for overcoming these hurdles. It emphasizes the imperative for continued research efforts and collaborative initiatives to fully harness the potential of ZnO NPs in developing advanced healthcare solutions with augmented antimicrobial and UV protective attributes. By advancing our understanding and leveraging innovative approaches, ZnO NPs hold promise for addressing pressing healthcare needs and enhancing patient care outcomes.

## Abstract 2

This paper presents a comprehensive review of recent advancements in utilizing zinc oxide nanoparticles (ZnO NPs) to enhance antimicrobial and UV protective properties in healthcare solutions.

## Graphical abstract

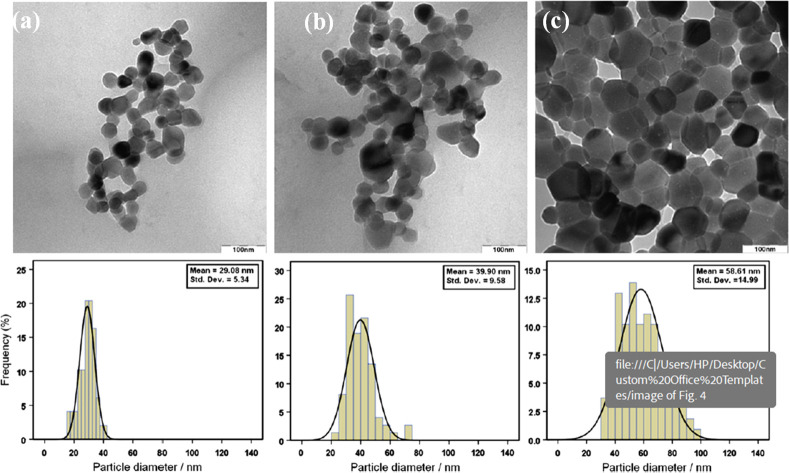

## 1 Introduction

In today's healthcare landscape, the need for effective antimicrobial solutions and robust UV protection has become increasingly paramount. The emergence of drug-resistant pathogens and the rising incidence of skin cancer underscore the urgency for innovative approaches to combat microbial infections and mitigate the harmful effects of ultraviolet (UV) radiation.^1–4^ In this context, zinc oxide nanoparticles (ZnO NPs) have emerged as versatile agents offering a dual advantage: potent antimicrobial activity and efficient UV shielding properties.

ZnO nanoparticles possess unique physicochemical properties that render them highly effective against a broad spectrum of microorganisms, including bacteria, viruses, and fungi.^5–7^ Their inherent antimicrobial efficacy arises from several mechanisms, such as the generation of reactive oxygen species (ROS) upon exposure to light and the disruption of microbial cell membranes. Furthermore, ZnO NPs exhibit exceptional stability, biocompatibility, and low toxicity, making them attractive candidates for various biomedical applications.^8,9^

The integration of ZnO nanoparticles into healthcare solutions holds significant promise for enhancing antimicrobial and UV protective properties.^10,11^ By harnessing the synergistic benefits of ZnO NPs, it becomes possible to develop advanced materials and formulations capable of combating infections and minimizing UV-induced damage simultaneously.^12–14^ Whether incorporated into wound dressings, medical textiles, or sunscreen formulations, ZnO NPs offer a multifaceted approach to addressing key healthcare challenges.

The aim of this study is to provide a comprehensive review of recent advancements in utilizing zinc oxide nanoparticles (ZnO NPs) to enhance antimicrobial and UV protective properties in healthcare solutions. This includes delving into the synthesis techniques of ZnO NPs, elucidating their antimicrobial efficacy, exploring the underlying mechanisms governing their action against a spectrum of pathogens, and analyzing factors impacting their antimicrobial performance. Furthermore, the study critically examines recent studies showcasing the effectiveness of ZnO NPs against diverse pathogens and investigates their UV protective capabilities, mechanisms, recent innovations in UV-blocking applications, and factors influencing UV shielding performance. Additionally, the paper addresses challenges associated with integrating ZnO NPs into healthcare products and presents future perspectives for overcoming these hurdles. The overarching goal is to emphasize the imperative for continued research efforts and collaborative initiatives to fully harness the potential of ZnO NPs in developing advanced healthcare solutions with augmented antimicrobial and UV protective attributes, ultimately enhancing patient care outcomes.

## 2 Synthesis methods of zinc oxide nanoparticles

Zinc oxide nanoparticles (ZnO NPs) are synthesized using a variety of methods, each offering unique advantages and considerations. This comprehensive review delves into the diverse synthesis techniques employed to produce ZnO NPs, providing a detailed analysis of their scalability, cost-effectiveness, and purity levels. The synthesis of ZnO nanoparticles encompasses a wide array of methodologies, including but not limited to sol–gel synthesis, hydrothermal synthesis, green synthesis, mechanochemical methods, and vapor-phase synthesis.^15,16^ Each of these approaches presents distinct characteristics and challenges that impact the efficiency, reproducibility, and properties of the resulting ZnO nanoparticles.

The selection of the appropriate synthesis method plays a critical role in tailoring the size, morphology, crystallinity, and surface properties of ZnO NPs to fulfill specific application requirements across various fields such as catalysis, sensing, biomedicine, and optoelectronics.^17–19^ Furthermore, optimizing synthesis parameters and post-synthesis treatments are essential steps in enhancing the performance and functionality of ZnO nanoparticles for advanced technological applications. The continuous exploration and advancement of synthesis techniques for ZnO nanoparticles are vital in unlocking their full potential for addressing diverse challenges in public health, environmental remediation, and cutting-edge technological innovations.^20–22^ In this study, the focus will be on sol–gel synthesis, hydrothermal synthesis, and green synthesis methods.

### 2.1 Sol–gel synthesis

Sol–gel synthesis enables precise control over particle size and morphology through the manipulation of precursor concentrations and reaction conditions. This method involves the hydrolysis and condensation of metal alkoxides to form a sol, followed by gelation and drying to obtain ZnO nanoparticles. Sol–gel synthesis offers high purity and control over particle characteristics, making it suitable for applications requiring tailored properties.^23,24^

**Fig. 1:**
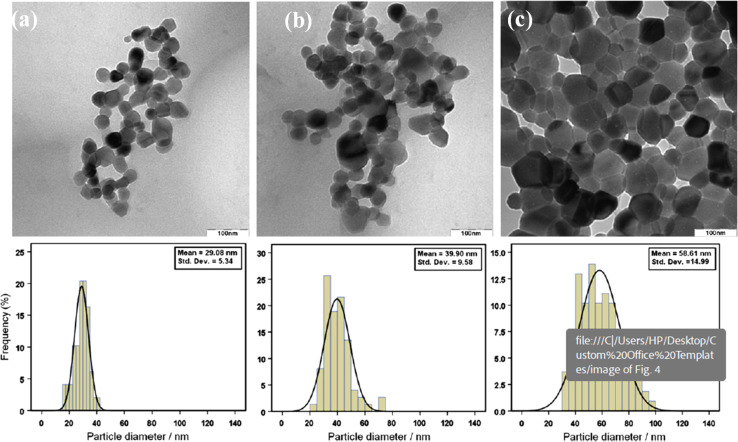
TEM images of ZnO-NPs prepared at different annealing temperatures: (a) 500, (b) 600 and (c) 700 °C.^25^

The synthesis of zinc oxide nanoparticles (ZnO-NPs) *via* the sol–gel method in gelatin media, as conducted by Zak *et al.* (2011), yielded nanoparticles with distinct properties influenced by the synthesis conditions, particularly the calcination temperature.^25^ Gelatin, being a long-chain compound, was employed both to terminate the growth of ZnO-NPs and to stabilize them during the synthesis process. Characterization of the synthesized ZnO-NPs was conducted through various techniques, including X-ray diffraction analysis (XRD), Fourier transform infrared spectroscopy (FTIR), and high-magnification transmission electron microscopy (TEM). The obtained results provided insights into the morphology, size distribution, and crystalline structure of the nanoparticles. As observed from the TEM images (Fig. 1), the morphology of the ZnO-NPs varied with the calcination temperature. At lower calcination temperatures (500 °C and 600 °C), both hexagonal and circular shapes were exhibited by the nanoparticles. However, as the calcination temperature increased to 700 °C, the nanoparticles predominantly displayed a hexagonal shape. This transition to hexagonal morphology was accompanied by an increase in the number of hexagonally shaped nanoparticles, which correlates with the growth in particle size. The size histograms constructed from the TEM images indicated an increase in the main particle size of the ZnO-NPs with increasing calcination temperature (Fig. 1). Specifically, the average particle sizes were approximately 29 ± 5 nm, 40 ± 10 nm, and 58 ± 15 nm for calcination temperatures of 500 °C, 600 °C, and 700 °C, respectively. This size evolution suggests that the calcination temperature plays a significant role in controlling the growth and size distribution of ZnO-NPs synthesized *via* the sol–gel method. Furthermore, the XRD analysis confirmed that the ZnO-NPs exhibited a hexagonal (wurtzite) structure across all calcination temperatures, indicating the preservation of crystalline integrity during synthesis. The observed variations in morphology and size distribution underscore the importance of understanding and controlling synthesis parameters, such as temperature, to tailor the properties of ZnO-NPs for specific applications. Overall, the study highlights the effectiveness of gelatin as a stabilizer in the sol–gel synthesis of small ZnO-NPs and underscores the influence of calcination temperature on the morphology and size of the synthesized nanoparticles. These findings contribute to the growing body of knowledge regarding the synthesis and manipulation of ZnO-NPs for various biomedical and technological applications.

Jurablu *et al.* (2015) employed the sol–gel method to synthesize zinc oxide (ZnO) nanoparticles, utilizing zinc sulfate heptahydrate dissolved in an ethanol solution containing diethylene glycol surfactant.^26^ Their investigation revealed the formation of ZnO nanoparticles with a hexagonal wurtzite structure, as confirmed by X-ray diffraction (XRD) analysis. Notably, the average particle size was approximately 28 nm, indicating finely scaled nanodimensions. Microstructural examination through scanning electron microscopy (SEM) and transmission electron microscopy (TEM) revealed spherical nanoparticles, often prone to clustering, suggesting potential aggregation tendencies. Fourier transform infrared spectroscopy (FTIR) spectra exhibited characteristic absorption peaks attributed to ZnO nanoparticles, verifying their purity. The determination of the wide bandgap energy at 3.49 eV further highlighted the optical properties of the synthesized nanoparticles.

X-ray diffraction (XRD) was utilized to identify crystalline phases and estimate crystalline sizes. The diffraction peaks corresponded to the reflection from various crystal planes of the hexagonal wurtzite zinc oxide structure. The mean size of the ordered ZnO nanoparticles was estimated to be around 25 nm. Field emission scanning electron microscopy (FE-SEM) analysis demonstrated increased homogeneity in the sample surface with increasing annealing temperature. The morphology of the particles changed to a spherical shape, and nanopowders were less agglomerated at higher temperatures. Transmission electron microscopy (TEM) analysis confirmed the actual size of the particles, their growth pattern, and the distribution of the crystallites. The TEM image revealed ZnO nanoparticles with an average diameter of about 28 nm. FT-IR spectra of the ZnO powders exhibited characteristic absorption peaks, consistent with the XRD results.

UV-Vis absorption spectra of both as-prepared and annealed ZnO nanoparticles indicated strong absorption bands at low wavelengths, with the absorption edge extending to longer wavelengths for annealed nanoparticles. The absorption positions depended on the morphologies and sizes of ZnO, with the UV absorption ability related to the band gap energy.

Alwan *et al.* (2015) conducted the synthesis of zinc oxide (ZnO) nanoparticles using the sol–gel method, with zinc acetate employed as a precursor.^27^ Their primary focus was on characterizing the crystalline structure and morphology of the synthesized nanoparticles. X-ray diffraction (XRD) analysis confirmed a highly crystalline wurtzite structure for the ZnO nanoparticles, consistent with previous findings. Additionally, Fourier transform infrared (FT-IR) spectra depicted characteristic absorption bands associated with ZnO nanoparticles, further confirming their chemical composition.

SEM revealed spherical nanoparticles with smooth surfaces, indicating uniformity in particle morphology. XRD patterns exhibited Bragg reflections corresponding to various crystal planes of ZnO, confirming the hexagonal phase of the nanoparticles. The average particle size was determined as 58.3 nm from the width of dominant peaks in the XRD pattern, in line with the Debye–Scherrer equation.

FT-IR spectroscopy provided additional evidence of the high purity of the synthesized ZnO nanoparticles, with characteristic absorption peaks corresponding to Zn–O bonds and hydroxyl groups. SEM images showed spherical ZnO particles with smooth surfaces, ranging in size from 100–200 nm. Furthermore, coating freshly prepared ZnO sol–gel onto polyethylene thin film resulted in particle sizes of approximately 50–60 nm. Inductively coupled plasma optical emission spectrometry (ICP-OES) elemental analysis confirmed the high yield of ZnO nanoparticles, with a purity of about 98.2%. UV-Vis optical absorption spectra exhibited a sharp absorbance onset at 345 nm, indicating almost uniform nanoparticle size. The optical band gap of the sol samples was estimated from UV-Vis Diffuse Reflectance Spectroscopic (UV-Vis DRS) studies, revealing a direct band gap of 2.935 eV. Alwan *et al.* (2015)^27^ successfully synthesized ZnO nanoparticles *via* the sol–gel method and comprehensively characterized their crystalline structure, morphology, and optical properties. Their findings contribute valuable insights into the synthesis and characterization of ZnO nanoparticles, with implications for various applications in nanotechnology and materials science.

Balcha *et al.* (2016) conducted a comparative study on the synthesis of zinc oxide (ZnO) nanoparticles using precipitation and sol–gel methods.^28^ Their research focused not only on the structural characterization of the synthesized nanoparticles but also on evaluating their photocatalytic activity. Both synthesis routes produced ZnO nanoparticles with a hexagonal wurtzite structure, confirmed by X-ray diffraction (XRD) patterns. However, the sol–gel method resulted in slightly smaller crystallite sizes compared to the precipitation method.

The XRD patterns of the ZnO powders synthesized by both methods showed identical diffraction peaks at specific angles (2*θ* = 31.8, 34.5, 36.4, 47.6, 56.8, and 63.0), corresponding to the crystal planes (100), (002), (101), (102), (110), and (103) respectively. These peaks confirmed the hexagonal wurtzite structure of the nanoparticles. The absence of additional diffraction peaks indicated the purity of the synthesized ZnO samples. The XRD results also showed that the (101) plane was the preferred growth plane, as indicated by the most intense peak, and the intensity ratio of the (101) to (002) peaks remained invariant, suggesting that solvent viscosity did not affect ion diffusion during synthesis. The average crystallite size of the synthesized photocatalysts was determined using Scherrer's equation, which considers the shape factor, wavelength of X-rays, full width at half maximum (FWHM) of the most intense diffraction peak, and Bragg's angle. The calculated average crystallite sizes of the ZnO nanoparticles synthesized *via* both methods were recorded and compared.

Crucially, the study found that ZnO nanoparticles synthesized *via* the sol–gel method exhibited superior photocatalytic activity compared to those synthesized *via* precipitation. This enhanced photocatalytic performance was attributed to the better colloidal stabilization of the nanoparticles produced by the sol–gel method. The smaller crystallite size and improved dispersion of these nanoparticles likely contributed to their increased effectiveness in photocatalytic applications.

In general, Balcha *et al.* (2016)^28^ demonstrated that while both the precipitation and sol–gel methods can produce pure ZnO nanoparticles with similar structural properties, the sol–gel method offers advantages in terms of producing smaller particles with enhanced photocatalytic activity, making it a more suitable technique for applications requiring high-performance ZnO nanoparticles.

Hasnidawani *et al.* (2016)^29^ conducted an insightful study focused on synthesizing zinc oxide (ZnO) nanoparticles using the sol–gel method, with zinc acetate dehydrate serving as the precursor. This study employed a suite of comprehensive characterization techniques, including X-ray diffraction (XRD), energy-dispersive X-ray spectroscopy (EDX), and field emission scanning electron microscopy (FE-SEM). The EDX analysis confirmed the high purity of the synthesized ZnO nanoparticles, indicating a zinc content of 55.38% and oxygen at 44.62%, which closely aligns with theoretical expectations. These results indicate minimal impurities, validating the effectiveness of the synthesis method.

XRD diffractogram revealed characteristic peaks of ZnO, affirming its crystalline nature. The patterns were consistent with standard data (JCPDS card no 36-1451), showing sharp peaks that signify good crystallinity. Notably, the highest intensity peaks were observed at 33.8616° and 36.2695°, corresponding to the (101) and (103) planes, respectively. FE-SEM imaging provided detailed insights into the morphology of the nanoparticles, which exhibited uniform rod-like structures. The homogeneous size distribution further emphasized the consistency of the synthesis process.

Particle size analysis, supported by nano particle analyzer results, confirmed that the ZnO nanoparticles had an average size of approximately 84.98 nm. Differential Scanning Calorimetry (DSC) was utilized to explore the thermal behaviour of the ZnO nanoparticles. The DSC thermograph displayed endothermic peaks primarily due to ethanol vaporization, with a minor exothermic peak at 26 °C, likely caused by changes in heat transfer dynamics and vapor pressure effects within the sample pan.

In summary, while all studies successfully synthesized ZnO nanoparticles *via* the sol–gel method, variations in precursor materials, surfactants, and reaction conditions led to discernible differences in nanoparticle properties. Notable variations encompassed particle size, morphology, crystallinity, and purity. The studies by Jurablu *et al.* (2015)^26^ and Alwan *et al.* (2015)^27^ underscored structural characterization and optical properties, emphasizing the significance of synthesis parameters. Balcha *et al.* (2016)'s study introduced a comparative perspective by evaluating photocatalytic activity, highlighting the importance of synthesis route selection for specific applications.^28^ Hasnidawani *et al.* (2016)'s study provided comprehensive characterization, further showcasing the sol–gel method's efficacy in producing ZnO nanoparticles with desirable properties.^29^

**Fig. 2:**
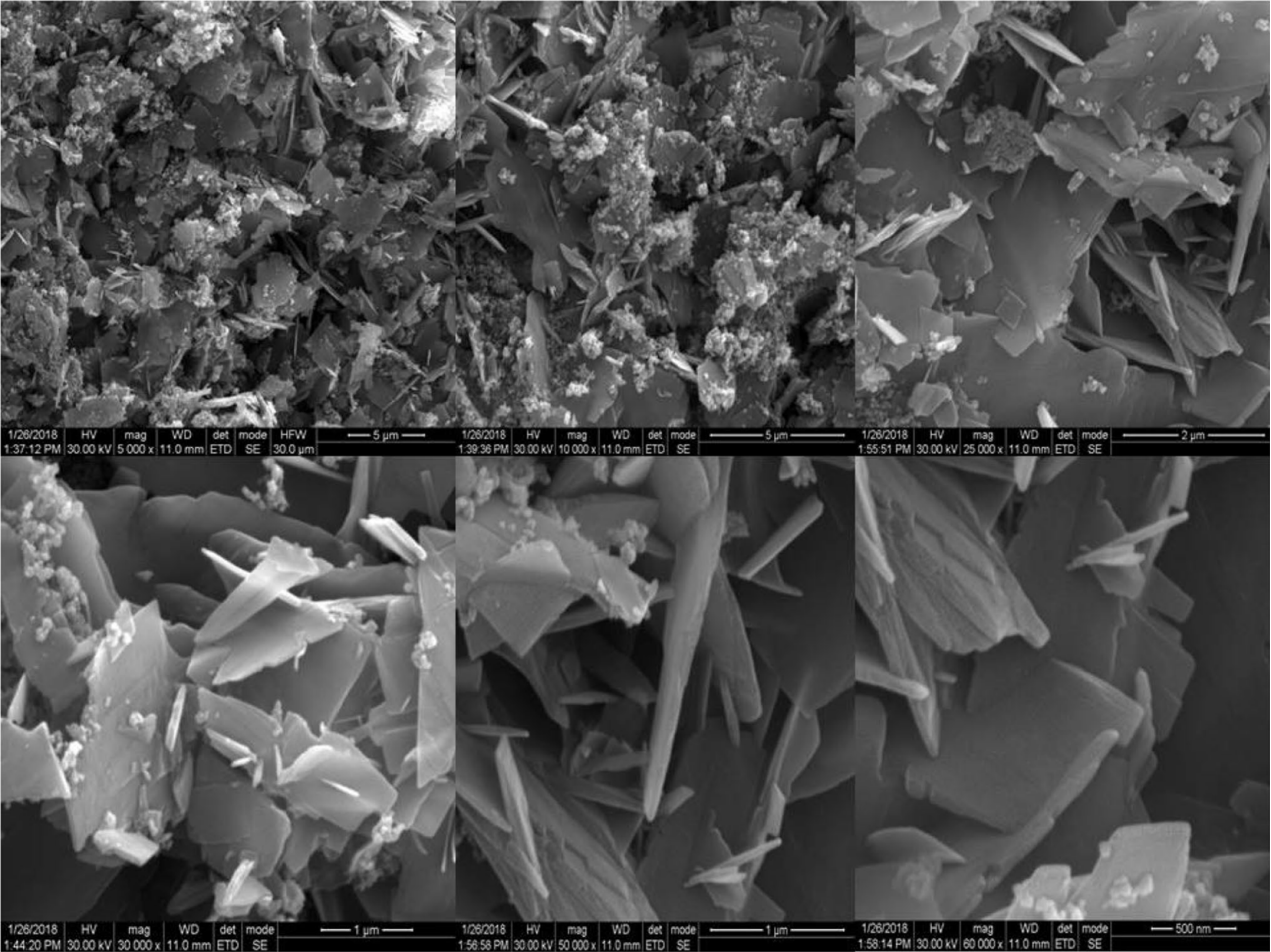
FE-SEM morphology of ZnO nanoflakes.^30^

Manikandan *et al.* (2018) conducted a study on the synthesis and properties of zinc oxide (ZnO) nanoparticles *via* the sol–gel method using zinc acetate as a precursor.^30^ The synthesized nanoparticles underwent extensive characterization through various analytical techniques. X-ray diffraction (XRD) analysis revealed that the synthesized ZnO nanoparticles exhibited a single phase with a wurtzite hexagonal structure. The lattice parameters were estimated using the Scherrer formula, and microstrain, stress, energy density, and crystallite size were analyzed using the Williamson–Hall model. The nanoparticles displayed well-defined crystalline characteristics. Fourier transform infrared spectroscopy (FTIR) spectra showed characteristic absorption peaks at 458.82 cm^−1^, confirming the presence of ZnO nanoparticles in the sample. Field emission scanning electron microscopy (FE-SEM) images revealed a flaky morphology of the synthesized ZnO nanopowder, with flat and irregularly shaped nanocrystalline flakes (Fig. 2).

**Fig. 3:**
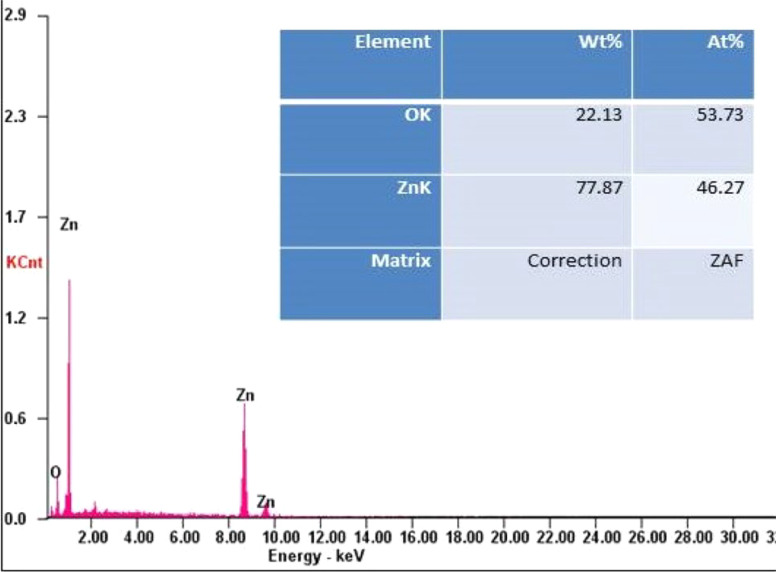
EDX spectrum of ZnO nanoflakes.^30^

Energy dispersive X-ray analysis (EDX) confirmed the presence of high-purity ZnO nanoparticles, with elemental composition comprising mainly zinc and oxygen (Fig. 3). The optical band gap of the nanoparticles was determined to be 3.1 eV through UV-visible spectroscopy. Raman spectroscopy further validated the chemical composition by confirming the presence of Zn–O stretching modes. The electrical properties of the ZnO nanoparticles, including dielectric constant, dielectric loss, and AC conductivity, were analyzed using impedance data. Additionally, the nanoparticles exhibited excellent photocatalytic behavior, with a calculated rate constant of 0.0296 min^−1^, as evaluated through methylene blue dye degradation studies.

In summary, Manikandan *et al.* (2018).'s study demonstrates the successful synthesis of ZnO nanoparticles *via* the sol–gel method and provides a detailed characterization of their structural, morphological, chemical, optical, and electrical properties.^30^ These findings contribute to the understanding of ZnO nanoparticles' fundamental characteristics for potential applications in various fields.

In another study, Mikhnev *et al.* (2019) utilized the sol–gel method employing zinc acetate in water to synthesize nano-sized zinc oxide (ZnO).^31^ This approach is widely recognized for its simplicity, scalability, and potential to produce nanoparticles with controlled size and morphology. By employing a sol–gel route, researchers can effectively control the nucleation and growth of nanoparticles, leading to tailored properties suitable for various applications.

The structural features of the synthesized ZnO samples were extensively characterized, focusing on their spectral features of luminescence and photoconductivity. This characterization provided valuable insights into the optical and electronic properties of the synthesized nanoparticles. Furthermore, the influence of treatment temperature on the luminescence and photoconductivity of nano-sized zinc oxide was systematically investigated. This aspect is crucial as thermal treatment can significantly alter the crystallinity and morphology of the nanoparticles, thereby affecting their optoelectronic properties.

**Fig. 4:**
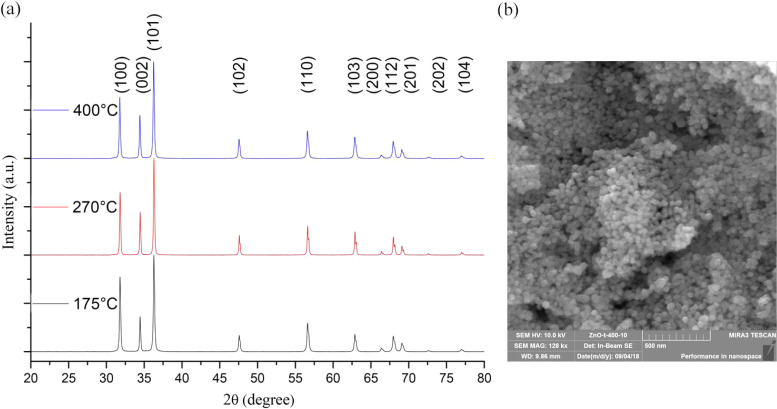
XRD patterns of ZnO samples annealed at different temperatures (a) and an electron microscopic image of a ZnO sample subjected to thermal treatment at a temperature of 400 °C (b).^31^

As depicted in Fig. 4, the diffractograms of the ZnO samples subjected to thermal treatment at different temperatures exhibited characteristic peaks associated with zinc oxide in the wurtzite-type structure. The observed increase in peak intensity and narrowing with higher calcination temperatures indicated an enhancement in crystallinity, attributed to the growth and aggregation of ZnO nanoparticles during sintering. These findings align with the Debye–Scherrer formula analysis, which estimated the crystallite size for the (101) plane. Notably, samples subjected to higher calcination temperatures exhibited larger crystallite sizes, highlighting the influence of thermal treatment on nanoparticle dimensions. Electron-microscopic analysis revealed that the synthesized zinc oxide nanoparticles exhibited a spherical shape, as depicted in Fig. 3. This morphology, close to spherical, is desirable for various applications due to its uniformity and high surface area-to-volume ratio, which can enhance reactivity and performance.

Sikdar *et al.* (2020)^32^ and Vishwakarma (2020)^33^ both address the synthesis of ZnO nanoparticles *via* the sol–gel method but with different emphases. Sikdar *et al.* (2020) specifically investigate the application of sol–gel-synthesized nano ZnO as a cure activator in rubber composites, highlighting its superior performance compared to conventional ZnO.^32^ In contrast, Vishwakarma (2020) provides a more generalized overview of ZnO nanoparticle synthesis using the sol–gel method, focusing on the method's advantages and the controlled synthesis of homogeneous ZnO nanoparticles with good crystallinity.^33^ While Sikdar *et al.* (2020) underscores the practical application of sol–gel-synthesized ZnO nanoparticles in rubber composites,^32^ Vishwakarma (2020) offers insights into the broader scope of the sol–gel method for nanoparticle fabrication.^33^

Singh *et al.* (2021) explored the synthesis of zinc oxide (ZnO) nanoparticles *via* the sol–gel method, focusing particularly on the characterization of structural and surface morphology.^34^ Utilizing zinc acetate dihydrate as a precursor, they successfully produced ZnO nanoparticles through this method. The study revealed significant insights into the effects of calcination temperature on particle and crystallite size.

SEM analysis indicated a change in average particle size, and crystallite size was calculated using the Debye–Scherrer equation based on the XRD peak for the (101) plane. As the calcination temperature increased from 500 °C to 900 °C, the crystal size increased while the overall particle size decreased. This suggests that higher temperatures reduce the crystal volume within the particles. Careful examination of the SEM images showed that particles were strongly agglomerated at lower calcination temperatures. Increasing the temperature reduced agglomeration, resulting in a higher surface area to volume ratio. This decrease in average particle size with increasing temperature was attributed to the reduction in particle agglomeration due to the high temperatures.

The experimental procedure employed by Singh *et al.* (2021)^34^ was noted for being cost-effective in producing homogeneous nanoparticles. The study suggests potential future research could involve modifying other physical parameters of the sol–gel process, such as pH, stirring time, and temperature, to observe the resultant effects on nanoparticle characteristics. Additionally, further adjustments to the synthesis and properties of nanoparticles could be explored, providing a broad scope for future investigations in this area.

The approach of Singh *et al.* (2021)^34^ complemented the findings of Sikdar *et al.* (2020)^32^ and Vishwakarma (2020)^33^ by emphasizing the structural properties of sol–gel-synthesized ZnO nanoparticles, thereby contributing to a comprehensive understanding of their characteristics.

In addition, the studies by Junaid (2022),^35^ Vignesh *et al.* (2022),^36^ Al-Shehri *et al.* (2023),^37^ and Madhuri (2023)^38^ collectively provide insights into the synthesis, characterization, and potential applications of zinc oxide (ZnO) nanoparticles.

Junaid (2022)^35^ and Vignesh *et al.* (2022)^36^ both investigated the synthesis of ZnO nanoparticles using the sol–gel method, utilizing different precursors and characterizing the resulting nanoparticles through a range of techniques to understand their structural, morphological, and optical properties. Junaid (2022) employed X-ray diffraction (XRD) at 40 kV to identify the crystalline phases and estimate the crystalline sizes of ZnO nanoparticles.^35^ The XRD patterns of the as-prepared and annealed samples at 500 °C reveal diffraction peaks corresponding to the hexagonal wurtzite structure of ZnO. The mean size of the ZnO nanoparticles is calculated to be around 25 nm using the Debye–Scherrer equation.

Field emission scanning electron microscopy (FE-SEM) is used to study the morphology of the ZnO nanoparticles, showing that increasing the annealing temperature results in high homogeneity and changes the particles to a more spherical shape with reduced agglomeration. SEM images indicate that the crystallite size of the annealed nanocrystals ranges from 20 to 80 nm in diameter. Transmission electron microscopy (TEM) analysis further confirms the particle size, showing an average diameter of about 28 nm for the as-synthesized ZnO nanoparticles. Fourier-transform infrared spectroscopy (FT-IR) spectra of the ZnO powders show characteristic peaks for OH groups, H–O–H bending vibrations, and Zn–O stretching vibrations, aligning well with the XRD results. UV-Vis absorption spectra indicate strong absorption bands near 355 nm for as-synthesized ZnO nanoparticles and near 410 nm for annealed samples, suggesting a phase transition influenced by heat treatment and highlighting the dependence of UV absorption properties on the nanoparticles' morphologies and sizes.

Vignesh *et al.* (2022) synthesize pure ZnO nanoparticles using the sol–gel method and characterize the powdered samples through X-ray diffraction (XRD) to determine their crystallinity.^36^ The XRD analysis indicates an average crystalline size of 36 nm, calculated using the Scherrer equation based on the peak widths at the (100) and (101) planes. SEM images revealed the ZnO nanoparticles forming flocks with various shapes. The size observed in SEM is larger than that obtained from XRD, suggesting that SEM measures the size of the entire flocks rather than individual crystals.

Overall, both studies demonstrated that the sol–gel method is effective for synthesizing ZnO nanoparticles with high crystallinity and distinct morphological characteristics. Junaid (2022)^35^ provides a detailed analysis of the structural changes and optical properties influenced by annealing, while Vignesh *et al.* (2022)^36^ focuses on the crystalline size and morphological variety observed in the SEM analysis. Together, these studies contributed to a deeper understanding of the properties of ZnO nanoparticles, which are crucial for their applications in various fields such as UV protection, sensors, and catalysis.

In contrast, Madhuri (2023) extended this analysis by comparing the sol–gel method with the hydrothermal method for synthesizing ZnO nanoparticles.^38^ The X-ray diffraction (XRD) analysis revealed that sol–gel synthesized nanoparticles have an average size of 14.36 nm, while hydrothermal synthesis produces mostly spherical nanoparticles with an average diameter of 14.5 nm. Scanning electron microscopy (SEM) indicates that both methods resulted in particle agglomeration, with sol–gel particles showing agglomeration at higher resolution and hydrothermal particles forming spherical aggregates. UV-visible spectroscopy showed that the sol–gel ZnO nanoparticles absorb UV radiation up to 362 nm and transmit most of the visible spectrum, whereas hydrothermal ZnO nanoparticles absorb UV radiation up to 368 nm.

The detailed findings highlighted the nuanced differences between the two synthesis methods in terms of nanoparticle size, shape, and optical properties. The sol–gel method yielded slightly smaller particles with high-resolution agglomeration, while the hydrothermal method produces slightly larger, spherical particles. Both types of nanoparticles exhibit similar UV absorption characteristics, making them suitable for applications requiring UV radiation absorption.

While Junaid (2022)^35^ and Vignesh *et al.* (2022)^36^ primarily emphasize the synthesis and characterization of ZnO nanoparticles, Al-Shehri *et al.* (2023)^37^ investigated the potential applications of Al-doped ZnO nanoparticles, particularly in optoelectronic devices. Al-Shehri *et al.* (2023) successfully synthesized zinc oxide (ZnO) nanocrystalline powders with varying aluminum (Al) concentrations (ranging from 0 to 4 wt%) using the sol–gel technique. The structure and morphology of the aluminum-doped ZnO (AZO) nanoparticles were investigated through X-ray powder diffraction (XRD) and scanning electron microscopy (SEM). XRD analysis indicated a reduction in crystallite size as the Al doping ratio increased, with all samples exhibiting the ZnO phase without any additional peaks. Furthermore, UV-visible diffuse reflectance spectroscopy was employed to examine the impact of Al doping on the optical properties of the ZnO nanopowder.

The study concluded that increasing the Al concentration resulted in a decrease in the energy gap (*E*_g_) value, from 3.30 eV for undoped ZnO to 3.25 eV for AZO with the highest concentration of 4 wt%. This finding suggests a modification of the bandgap due to Al doping. Overall, the ability to tune the bandgap of the prepared samples makes them promising candidates for various applications, particularly in optoelectronic devices.

The reviewed studies collectively offered valuable insights into the synthesis, characterization, and potential applications of zinc oxide (ZnO) nanoparticles. By comparing different synthesis methods, doping strategies, and characterization techniques, they contribute to a deeper understanding of how to tailor the properties of ZnO nanoparticles for diverse applications.

### 2.2 Hydrothermal synthesis

Hydrothermal synthesis is a method of choice in nanomaterial synthesis, renowned for its ability to produce zinc oxide nanoparticles (ZnO NPs) with exceptional properties. This intricate process involves precisely controlling a chemical reaction between a zinc precursor and a hydrothermal solution under specific conditions of elevated temperatures and pressures.^39–41^ By adjusting parameters such as temperature, pressure, reaction duration, and precursor concentrations, researchers can intricately manipulate the nucleation and growth of ZnO crystals.^42^

One of the most striking advantages of hydrothermal synthesis lies in its unparalleled control over the size, shape, and structure of the resulting ZnO nanoparticles. This precise control enables researchers to tailor the properties of the nanoparticles to meet the exact requirements of diverse applications. Whether it's catalysis, sensors, biomedical devices, or optoelectronics, hydrothermal synthesis offers the versatility needed to fine-tune ZnO nanoparticles for optimal performance.^43,44^

In this method, the hydrothermal environment provides the ideal conditions for the nucleation and growth of ZnO crystals.^42^ The controlled reaction kinetics ensure the formation of nanoparticles with uniform size distribution and high crystallinity, essential for achieving desired functionalities.^43^ Moreover, the hydrothermal process allows for the formation of complex nanostructures, such as nanorods, nanowires, and nanosheets, which further expand the range of potential applications.^44^

Overall, hydrothermal synthesis stands as a cornerstone in the realm of nanomaterial fabrication, offering researchers a powerful tool to engineer ZnO nanoparticles with tailored properties and enhanced performance for a myriad of technological advancements and scientific endeavors. For example, Bazazi *et al.* (2018)^45^ and Marlinda *et al.* (2019)^46^ both explored the hydrothermal synthesis route but with variations in methodology and outcomes. Bazazi *et al.* (2018) investigated the synthesis of ZnO nanostructures using both hydrothermal and ball mill-hydrothermal methods.^45^ They found that ball milling before the hydrothermal process altered the morphology of the ZnO nanostructures, resulting in enhanced photocatalytic activity compared to conventional hydrothermal synthesis. This study underscores the importance of mechanical processing in modifying the properties of ZnO NPs. In contrast, Marlinda *et al.* (2019) focused on the one-step hydrothermal synthesis of ZnO nanostructures with varied morphologies by adjusting the pH level.^46^ They observed the formation of different ZnO morphologies, such as baton, star, flower, and rod-like structures, by varying the pH using sodium hydroxide (NaOH) as a base. The study highlights the influence of pH on the morphological properties of ZnO nanostructures and their photoelectrochemical (PEC) water-splitting properties.

Mahendiran *et al.* (2019)^47^ and Mohan *et al.* (2020)^48^ further contributed to the understanding of hydrothermal-synthesized ZnO NPs by focusing on their structural and optical properties. Mahendiran *et al.* (2019) synthesized nano-sized ZnO using a simple hydrothermal method and characterized the nanoparticles using various techniques, including X-ray diffraction (XRD), scanning electron microscopy (SEM), and UV-visible spectroscopy.^47^ Their findings provided insights into the crystalline structure, morphology, and bandgap of ZnO NPs synthesized *via* hydrothermal routes.

Similarly, Mohan *et al.* (2020) investigated the effects of reaction conditions on the properties of hydrothermally synthesized ZnO NPs.^48^ They observed changes in grain size, morphology, luminescence, and optical absorption of ZnO NPs with variations in reaction temperature and time. This study highlights the tunability of ZnO nanoparticle properties through hydrothermal synthesis parameters, offering valuable insights for tailoring ZnO-based materials for specific applications.

Furthermore, Chandrasekaran *et al.* (2020) explored a novel wet chemical hydrothermal synthesis method using natural extracts as solvent media for ZnO nanoparticle synthesis.^49^ They found that the choice of solvent media influenced the size, morphology, optical, and cytotoxic properties of ZnO NPs. This study demonstrates the potential of eco-friendly approaches for synthesizing ZnO NPs with enhanced antibacterial and anticancer properties.

In a different study, Gerbreders *et al.* (2020) investigated the hydrothermal synthesis method for producing zinc oxide (ZnO) nanostructures, a popular approach in nanomaterial synthesis.^50^ Utilizing a nitrate-based precursor reaction with equimolar amounts of hexamethylenetetramine (HMTA), the study aimed to elucidate the growth process and morphology control of ZnO nanostructures.

**Fig. 5:**
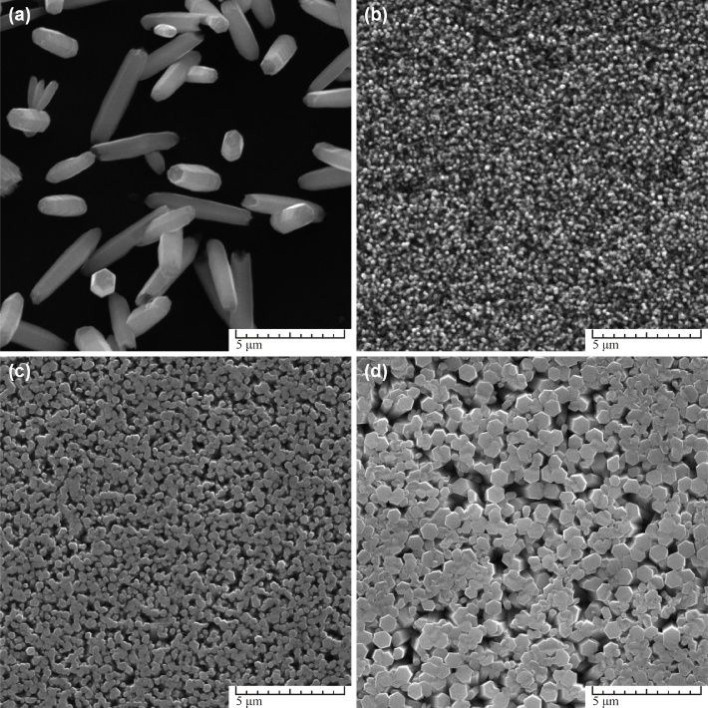
SEM images of ZnO nanostructures synthesized at different origin of seed layer: (a) on clear glass, (b) on MS seeds, (c) on ED seeds, (d) on AD seeds. Other parameters were: 0.1 M Zn(NO_3_)^2+^ HMTA equimolar solution, *t* = 3 h, *T* = 90 °C.^50^

In the absence of a seed layer, as depicted in Fig. 5a, nanostructures grown on clear glass substrates exhibited thick, disordered formations due to growth occurring mainly on surface defects. These defects, such as screw dislocations and rough edges, provided limited nucleation centres. Conversely, when seed layers were introduced, as shown in Fig. 7b–d, nanostructured coatings with a pronounced vertical arrangement of the nanostructures were produced, leading to homogeneous and dense coatings.

**Fig. 6:**
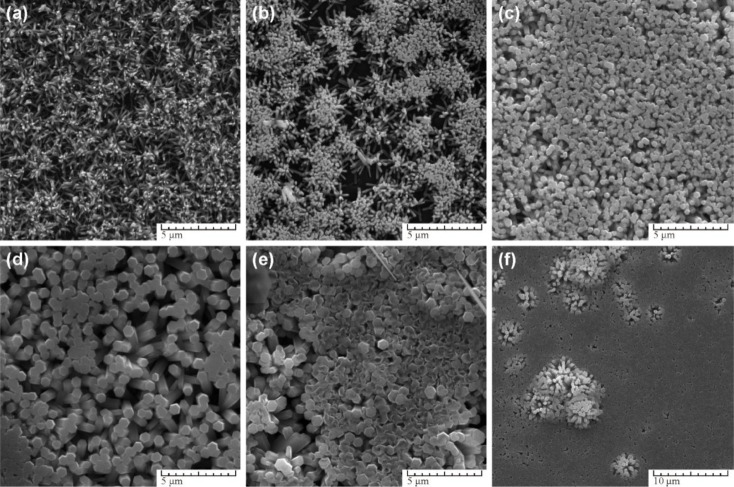
SEM images of ZnO nanostructures synthesized at different Zn(NO_3_)_2_^+^ HMTA solutions concentration: (a) 0.0125 M, (b) 0.025 M, (c) 0.05 M, (d) 0.1 M (e) 0.2 M, (f) 0.3 M. Other parameters were AD seeds, *t* = 3 h, *T* = 90 °C.^50^

The study explored the effect of solution concentration on nanostructure formation, as illustrated in Fig. 8. At lower concentrations (Fig. 6a), incomplete sharp-tipped rods were observed due to rapid depletion of the Zn^2+^ ion source. As the concentration increased (Fig. 6b and c), nanorods with flat surfaces formed, albeit with smaller diameters. Optimal diameter of nanorods was achieved at a concentration of 0.1 M (Fig. 6), beyond which distorted forms of nanostructures emerged. Furthermore, the research delved into the influence of synthesis parameters and capping agents on the growth process. By varying these parameters, the study identified optimal synthesis conditions for obtaining nine independent ZnO morphologies. The findings emphasized the production of durable, homogeneous epitaxial coatings on hard surfaces, presenting potential applications in sensor development and other fields where surface area is crucial.

Overall, the study contributes valuable insights into hydrothermal synthesis as a versatile method for producing tailored ZnO nanostructures with controlled morphologies, paving the way for diverse applications beyond conventional ZnO powders.

Qian (2022) presents a study on the synthesis of zinc oxide (ZnO) nanorods using an economical hydrothermal method.^51^ The research explores the formation mechanism of these nanoparticles and investigates the structural changes using various characterization techniques.

**Fig. 7:**
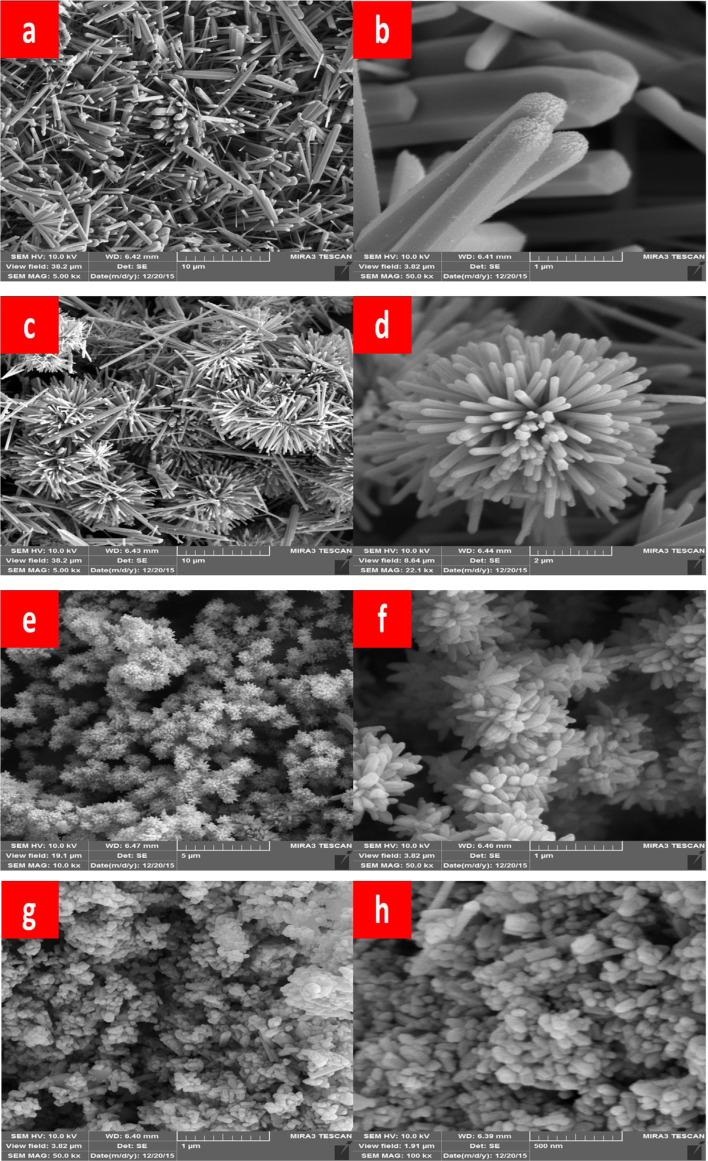
Typical FE-SEM prepared ZnO samples (a) and (b) hexagonal rods (ZnO-1), (c) and (d) flower-like rods (ZnO-2), (e) and (f) flowers-like prismatic (ZnO-3), (g) and (h) quasi-prismatic (ZnO-4).^51^

The synthesis method involves the use of a self-designed *in situ* temperature-pressure sample cell to control the hydrothermal conditions. The study examines the effect of different parameters such as the type of base (NaOH or KOH), ball milling, and temperature on the morphology of the ZnO nanoparticles. FE-SEM images reveal distinct morphologies for ZnO samples prepared with NaOH and KOH solutions, with variations observed in rod-like structures and flower-like microstructures (Fig. 7). Notably, ball milling is found to influence the morphology, leading to the formation of flower-like ZnO microstructures through the self-organization of pencil-like ZnO rods (Fig. 7).

**Fig. 8:**
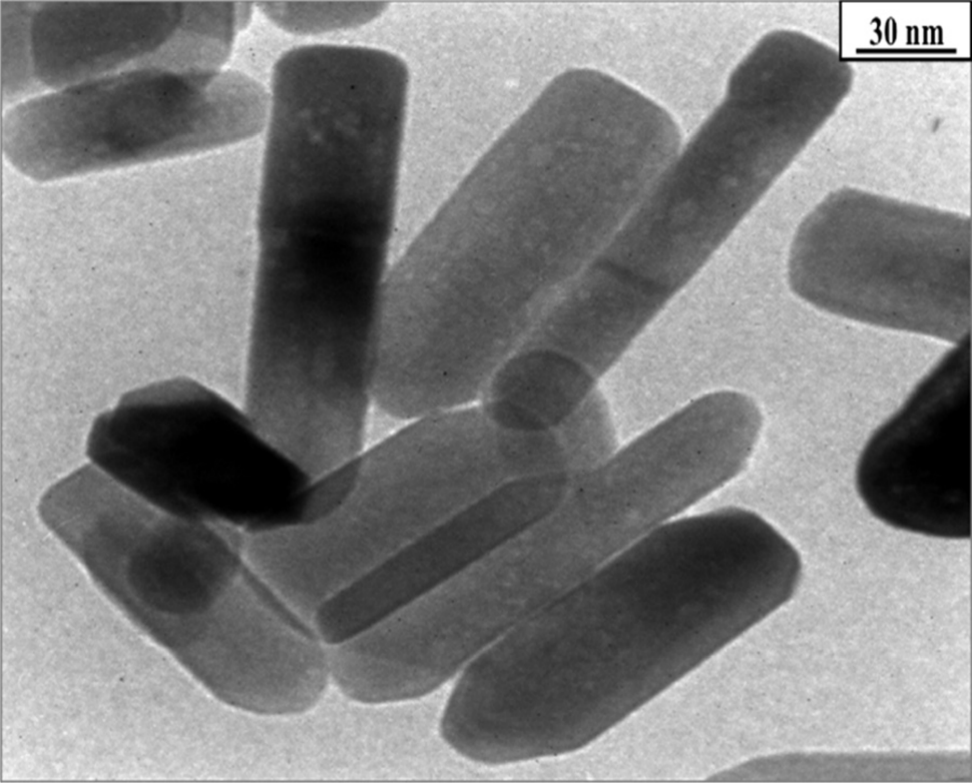
The transmission electron microscopy (TEM) image of ZnO-4 nanostructure.^51^

**Fig. 9:**
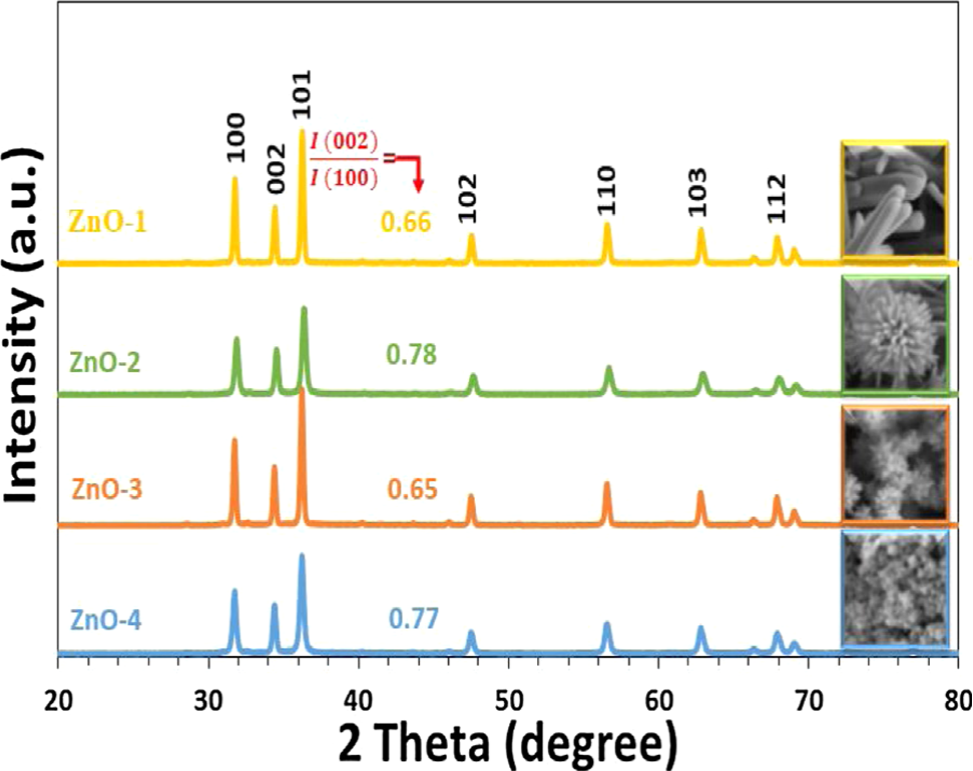
XRD patterns of prepared ZnO samples.^51^

The study also identifies an intermediate phase, Zn(HCO_3_)_2_·H_2_O, formed at lower temperatures, which undergoes transformation to ZnO nanorods at higher temperatures. XRD and TEM analyses confirm the wurtzite structure of the final ZnO nanorods, with average dimensions of 45 nm in diameter and 2 μm in length (Fig. 8 and 9).

Overall, the research sheds light on the formation mechanism of ZnO nanorods and provides insights for the controllable synthesis of ZnO nanoparticles, contributing to the advancement of nanomaterial synthesis techniques.

The research conducted by Wirunchit & Koetniyom (2022) focuses on the synthesis of zinc oxide (ZnO) nanoparticles *via* hydrothermal precipitation solutions for potential biological applications.^52^ They investigated the synthesis of ZnO nanoparticles using zinc nitrate (Zn(NO_3_)_2_) as the precursor and three different precipitator solutions: potassium hydroxide (KOH), sodium hydroxide (NaOH), and ammonium hydroxide (NH_4_OH). Various characterization techniques including X-ray diffraction (XRD), Fourier transform infrared spectroscopy (FT-IR), Raman spectroscopy, and field emission scanning electron microscopy (FE-SEM) were employed to analyze the structure, size, and morphology of the synthesized ZnO nanoparticles. Additionally, the antibacterial efficacy of the nanoparticles was studied. The results revealed that the smallest ZnO nanoparticles were obtained when using zinc nitrate reacted with sodium hydroxide as the precipitator at 120 °C for 4 h. This study highlights the importance of precursor and precipitator selection in tailoring the properties of ZnO nanoparticles for specific applications, particularly in biological contexts.

On the other hand, Seitov & Kazbek (2022) focused on the structural properties and morphology of zinc oxide (ZnO) thin films obtained through hydrothermal synthesis.^53^ They utilized ZnO nanofilms deposited by the sol–gel method as seed layers on the glass surface of STO (strontium titanate). The concentration of chemical reagents during the sol–gel transformation significantly influenced the properties of the resulting ZnO films. Through hydrothermal synthesis at approximately 97 °C for 3 hours, they successfully synthesized ZnO nanorods on the surface of the nanofilm. Subsequent firing of the synthesized ZnO nanorods led to a several-fold increase in electrical resistance. Characterization using scanning electron microscopy and X-ray diffraction revealed the chemical composition and morphology of the obtained nanorod arrays. Notably, the ZnO nanoparticle arrays exhibited enhanced response to visible light, suggesting potential applications in photoelectrochemical devices for solar energy conversion. These findings provide valuable insights for the development of high-performance photoelectrochemical systems and offer opportunities for further advancements in alternative energy technologies.

In summary, these studies collectively contribute to the understanding of hydrothermal synthesis methods for producing ZnO nanoparticles with tailored morphologies and properties. They underscore the importance of synthesis parameters, such as base type, pH level, reaction conditions, and solvent media, in controlling the structural, optical, and functional characteristics of ZnO nanostructures.

### 2.3 Green synthesis

Green synthesis of zinc oxide nanoparticles (ZnO NPs) has emerged as a promising and eco-friendly approach for the production of nanomaterials with various applications.^54^ Unlike traditional chemical methods that often involve hazardous chemicals and generate toxic by-products, green synthesis harnesses natural sources such as plant extracts, microorganisms, and biopolymers to facilitate the reduction and stabilization of metal ions into nanoparticles.^55,56^ This sustainable method not only reduces environmental impact but also offers several advantages including cost-effectiveness, scalability, and biocompatibility.^57,58^

One of the key advantages of green synthesis is its versatility, as it allows for the use of a wide range of biological materials as reducing and capping agents.^59,60^ Plant extracts, rich in phytochemicals such as polyphenols, flavonoids, and terpenoids, have been extensively explored for their potential in synthesizing ZnO NPs. These bioactive compounds possess inherent reducing properties that facilitate the conversion of metal ions into nanoparticles.^61^ Additionally, the phytochemicals present in plant extracts also serve as stabilizing agents, preventing the agglomeration and ensuring the stability of the synthesized nanoparticles.^62,63^

Microorganisms, including bacteria, fungi, and algae, have also demonstrated the ability to mediate the green synthesis of ZnO NPs. Microbial systems offer unique advantages such as rapid synthesis rates, high yields, and the ability to produce nanoparticles under mild reaction conditions.^64–66^ Moreover, the diverse metabolic pathways of microorganisms enable the synthesis of ZnO NPs with tailored properties, including size, shape, and crystallinity.^67^

Biopolymers, such as chitosan, cellulose, and starch, have garnered significant attention as green templates for the synthesis of ZnO NPs.^68^ These naturally occurring polymers provide a biocompatible and environmentally friendly matrix for the nucleation and growth of nanoparticles.^69^ Through interactions with metal ions, biopolymers facilitate the formation of ZnO NPs with controlled morphology and size distribution. Furthermore, the inherent biodegradability and non-toxic nature of biopolymers make them suitable for various biomedical and environmental applications.^70^

In addition to their eco-friendliness, ZnO NPs synthesized *via* green methods exhibit remarkable physicochemical properties that render them suitable for diverse applications. Their unique optical, electrical, and catalytic properties make them promising candidates for applications in photocatalysis, sensing, drug delivery, antimicrobial agents, and environmental remediation.^63–65^ Furthermore, the biocompatibility of green-synthesized ZnO NPs opens up avenues for their use in biomedical applications such as cancer therapy, bioimaging, and tissue engineering.^63^

Overall, green synthesis offers a sustainable and environmentally friendly approach for the production of ZnO nanoparticles with tailored properties and versatile applications. By harnessing the power of nature, green synthesis not only addresses the challenges associated with traditional synthesis methods but also paves the way for the development of innovative nanomaterials with enhanced performance and reduced environmental impact.

For example, Raja *et al.* (2018) described the green synthesis of Zinc Oxide Nanoparticles (ZnO NPs) utilizing an aqueous *Tabernaemontana divaricata* green leaf extract.^71^ The synthesized ZnO NPs underwent extensive characterization employing X-ray diffraction (XRD), Ultraviolet-Visible (UV-Vis) studies, Scanning Electron Microscopy (SEM), Transmission Electron Microscopy (TEM), and Fourier Transform-Infra Red (FT-IR) analysis.

**Fig. 10:**
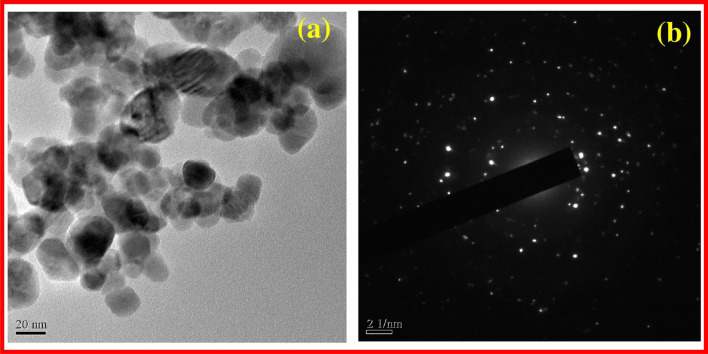
TEM image ZnO NPs (a) SAED pattern of ZnO NPs (b).^71^

XRD analysis confirmed the presence of a pure hexagonal wurtzite crystalline structure of ZnO, indicative of high crystallinity. TEM imaging showcased spherical ZnO NPs with sizes ranging from 20 to 50 nm. Notably, Fig. 10a depicts the TEM image revealing nanoparticles within this size range. Furthermore, the selected area electron diffraction (SAED) pattern, as depicted in Fig. 10b, exhibited concentric rings, indicating the highly crystalline nature of the synthesized ZnO NPs.

FT-IR analysis provided additional insights, suggesting that the obtained ZnO NPs were stabilized through interactions with various bioactive compounds present in the leaf extract, enhancing their stability and functionality.

In summary, Raja *et al.*'s^67^ study demonstrates the successful green synthesis of ZnO NPs using *Tabernaemontana divaricata* leaf extract. The comprehensive characterization reveals the high quality, crystallinity, and stability of the synthesized nanoparticles, highlighting their potential for diverse applications in biomedicine, catalysis, and environmental remediation.

**Fig. 11:**
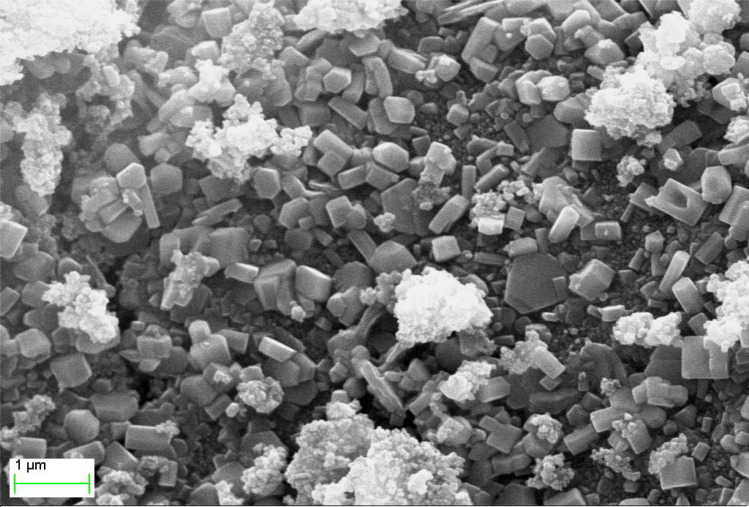
A FE-SEM image.^72^

Khatami *et al.* (2018) present a novel synthesis method for zinc oxide (ZnO) nanostructures using a natural sweetener extract from Stevia, which offers a fast, clean, and environmentally friendly approach.^72^ The synthesized ZnO nanoparticles exhibited predominantly rectangular morphology, with some instances of square, octagonal, and oval bar shapes, as depicted in Fig. 11*via* FE-SEM imaging.

Compositional analysis of the nanoparticles was conducted using energy-dispersive X-ray spectroscopy (EDX), confirming the presence of zinc and oxygen elements. The synthesized nanoparticles exhibited a rectangular shape with sizes ranging from 10 to 90 nm. Thermal stability of the rectangular-shaped ZnO nanoparticles was investigated through thermogravimetric analysis (TGA). The TGA and derivative thermogram revealed a slight sample weight loss of approximately 5.6%, attributed to the thermal degradation of biomolecules, such as Steviol glycosides present in the extract. However, the increment in temperature did not lead to significant weight loss, indicating high thermal stability of the ZnO nanoparticles.

Additionally, the zeta potential value of the ZnO nanoparticles was measured, revealing a negative zeta potential value of −25.1 mV. This information provides insights into the surface charge and stability of the synthesized nanoparticles.

Overall, Khatami *et al.*'s study demonstrates the efficient synthesis of ZnO nanoparticles using Stevia extract, highlighting their promising antimicrobial applications and offering valuable insights into their morphology, thermal stability, and surface properties.^72^

Kahsay *et al.* (2019) investigated the optical, structural, and morphological properties of ZnO nanostructures synthesized using a green approach.^73^ The synthesized nanostructures displayed a hexagonal wurtzite structure with an average particle diameter of 29 nm. These structural characteristics influence the optical properties and photocatalytic activity of the nanoparticles, demonstrating their potential for environmental remediation applications. Subsequently, Fakhari *et al.* (2019) demonstrated successful synthesis of ZnO nanoparticles using plant extracts, showcasing unique morphological features.^74^ The nanoparticles exhibited sizes ranging from 21.49 to 25.26 nm and an irregular spherical shape with an average size of 31 ± 2 nm, respectively. These variations in morphology could influence specific properties such as surface area and catalytic activity, illustrating the influence of green synthesis parameters on nanoparticle characteristics.

In a very recent study, Kiani *et al.* (2023) evaluated the structural and chemical properties of ZnO nanoparticles synthesized from *Citrullus colocynthis* extracts.^75^ The nanoparticles exhibited a spherical morphology with a size range between 64 and 82 nm and confirmed the presence of ZnO crystalline structure. Additionally, the nanoparticles demonstrated high purity and stability, indicating the effectiveness of the green synthesis approach in producing well-defined nanoparticles. Sánchez-Pérez *et al.* (2023) examined the impact of synthesis conditions on the characteristics of ZnO nanoparticles synthesized using *Larrea tridentata* extract.^76^ The nanoparticles exhibited a size range of 18 to 40 nm and demonstrated improved dispersibility and stability. These properties are essential for various applications, highlighting the effectiveness of green-synthesized nanoparticles in achieving desired characteristics. Al-Zahrani *et al.* (2023) investigated the structural and optical properties of ZnO nanoparticles synthesized from Punica granatum plant leaves extract.^77^ The nanoparticles exhibited a crystalline structure with a diameter of 20 nm and demonstrated high optical transparency. These properties are crucial for applications such as optoelectronic devices and photocatalysis, showcasing the potential of green-synthesized nanoparticles in achieving desirable properties for specific applications.

In summary, these studies collectively demonstrate the effectiveness of green synthesis approaches in tailoring the properties of ZnO nanoparticles. The diverse morphologies, structural features, and chemical compositions of the synthesized nanoparticles underscore the versatility and potential of green synthesis in nanomaterial fabrication for various applications.

## 3 Comparison of synthesis techniques for zinc oxide nanoparticles: advantages and 3.1 disadvantages

This review focuses on the sol–gel technique, hydrothermal synthesis, and green synthesis methods for synthesizing zinc oxide nanoparticles (ZnO NPs) due to their unique advantages and specific limitations. While many methods exist for ZnO NP synthesis, these three methods were selected for their relevance to various application needs and their distinct characteristics. However, it is essential to acknowledge the wide range of available synthesis techniques to provide a comprehensive understanding of their comparative benefits and drawbacks.

The sol–gel technique is favored for its ability to provide precise control over nanoparticle size and uniform particle size distribution. This precision makes it ideal for applications requiring well-defined nanoparticle characteristics, such as optoelectronics and sensors. However, the sol–gel method often necessitates high temperatures and the use of toxic chemicals, which can limit its environmental sustainability and increase costs. These requirements are also observed in methods such as chemical vapor deposition (CVD), microemulsion techniques, mechanochemical synthesis, precipitation methods, spray pyrolysis, electrochemical synthesis, sonochemical synthesis, template-assisted synthesis, flame spray pyrolysis, solvothermal synthesis, laser ablation, biomimetic synthesis, thermal decomposition, microwave-assisted synthesis, and combustion synthesis, all of which face similar environmental sustainability issues. Despite these drawbacks, the sol–gel method remains a valuable synthesis strategy due to its precision and reliability in producing high-quality nanoparticles.^78,79^

Hydrothermal synthesis is notable for its rapid production and high purity of ZnO NPs. This method is particularly advantageous for industrial-scale production due to its scalability. However, it operates under high-pressure conditions, which can pose safety challenges and require specialized equipment. Additionally, the control over particle shape in hydrothermal synthesis is relatively limited, which might impact its suitability for certain applications where specific nanoparticle morphology is crucial.^80,81^ These high-pressure conditions are also characteristic of the other mentioned methods, leading to similar limitations in controlling particle shape.

Green synthesis stands out for its environmentally friendly approach, utilizing plant extracts and other natural resources. This method is cost-effective and biocompatible, making it suitable for small-scale production and applications in healthcare and environmental sustainability. However, green synthesis can involve relatively longer synthesis times and variability in nanoparticle properties due to the natural variability of plant extracts, which can affect the consistency and reproducibility of the nanoparticles produced.^82^ This variability in synthesis time and properties is less prevalent in other methods such as CVD, microemulsion techniques, mechanochemical synthesis, precipitation methods, spray pyrolysis, electrochemical synthesis, sonochemical synthesis, template-assisted synthesis, flame spray pyrolysis, solvothermal synthesis, laser ablation, biomimetic synthesis, thermal decomposition, microwave-assisted synthesis, and combustion synthesis.^80,81^

Comparing these synthesis techniques reveals varying levels of cost-effectiveness and scalability. Green synthesis is particularly advantageous for its low environmental impact and suitability for biocompatible applications, albeit with some trade-offs in terms of synthesis time and consistency. The sol–gel method, while precise, faces challenges related to environmental sustainability and operational costs. Hydrothermal synthesis offers a balance of purity and scalability but with limitations in particle shape control and operational complexity. Table 1 specifically illustrates the comparison of synthesis techniques for zinc oxide nanoparticles in terms of their advantages and disadvantages.^78,79^

**Table 1:** Comparison of synthesis techniques for zinc oxide nanoparticles: advantages and disadvantages

Synthesis technique	Advantages	Disadvantages
Sol–gel method	(1) Precise control over nanoparticle size	(1) Requires high temperatures
	(2) Uniform particle size distribution	(2) May involve toxic chemicals
	(3) Versatile synthesis conditions	(3) Energy-intensive process
Hydrothermal synthesis	(1) Rapid synthesis	(1) High-pressure conditions
	(2) High purity of nanoparticles	(2) Limited control over particle shape
	(3) Scalable for industrial production	(3) Energy-intensive process
Green synthesis	(1) Environmentally friendly	(1) Relatively longer synthesis times
	(2) Cost-effective	(2) Variability in nanoparticle properties
	(3) Biocompatible	(3) Limited scalability for industrial use

Understanding the differences between these synthesis methods is crucial for tailoring ZnO NPs to meet specific application requirements, ensuring maximum efficacy and utility in various fields, including healthcare, antimicrobial formulations, and UV protection. This review aims to provide a comprehensive comparison of these methods, highlighting their respective advantages and limitations to guide researchers and practitioners in selecting the most appropriate synthesis strategy for their needs.

## 4 Antimicrobial properties of zinc oxide nanoparticles

Zinc oxide nanoparticles (ZnO NPs) have garnered significant attention for their antimicrobial properties, which make them promising candidates for various applications in healthcare, environmental remediation, and consumer products.^8,83^ The antimicrobial activity of ZnO NPs arises from their ability to induce oxidative stress, disrupt cell membrane integrity, and interfere with cellular functions in microorganisms.^84^

### 4.1 Mechanisms underlying the antimicrobial activity of ZnO NPs

The antimicrobial activity of ZnO nanoparticles (ZnO NPs) is driven by a multifaceted mechanism that encompasses several processes. Primarily, when exposed to light or in the presence of moisture, ZnO NPs exhibit the ability to generate reactive oxygen species (ROS) like hydrogen peroxide and superoxide radicals.^14^ These ROS serve as potent oxidative agents, initiating damage to microbial cell membranes, proteins, and genetic material (DNA), ultimately culminating in the demise of the microbial cells.^85^ For example, Kadiyala *et al.* (2018) investigated the fluorescence of ROS indicators, APF and H2DCFDA, in ZnO NP dispersions and H_2_O_2_ solutions.^86^ They observed a dose-dependent increase in fluorescence of both indicators in the presence of ZnO NPs, indicating the generation of ROS. However, the absolute fluorescence of APF for low-dose ZnO NPs was slightly lower than in pure water, suggesting potential saturation of APF on the NPs. In contrast, H2DCFDA showed less affinity for the NPs. Further experiments demonstrated a dose-dependent increase in ROS production by *S. aureus* exposed to ZnO NPs, albeit smaller than that induced by H_2_O_2_. The study also compared the ROS fluorescence required for a significant reduction in bacterial counts induced by H_2_O_2_ and ZnO NPs. It was found that H_2_O_2_ generated significantly greater amounts of ROS to achieve the same reduction in CFUs as ZnO NPs, suggesting a different mechanism of action than expected based solely on dose-dependent ROS generation.

Overall, the findings suggest that while ZnO NPs induce ROS production in microbial cells, the absolute amount of ROS generated is smaller than that produced by H_2_O_2_, leading to different conclusions about their antimicrobial mechanism.

**Fig. 12:**
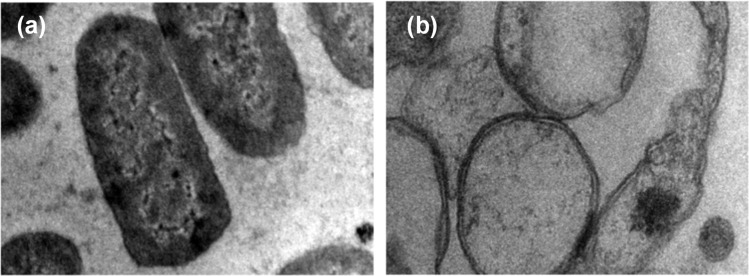
Transmission electron microscopy (TEM) image of *A. baumannii* cultured in the absence (a) and presence (b) of chemically synthesized ZnO-NPs.^87^

Moreover, the interaction between zinc oxide nanoparticles (ZnO NPs) and microbial cell membranes instigates a cascade of events that ultimately disrupts the structural integrity of the membranes. This direct engagement leads to perturbations in the membranes' architecture, resulting in compromised permeability and functionality. In a comprehensive investigation conducted by Tiwari *et al.* (2018), transmission electron microscopy (TEM) was employed to examine the effect of ZnO on bacterial cell membrane integrity (as depicted in Fig. 12).^87^ The TEM analysis delineated a stark contrast between the membranes of bacteria exposed to ZnO and those under control conditions. In the absence of ZnO treatment (control condition), the bacterial membranes appeared intact and structurally sound (Fig. 12a). However, upon exposure to ZnO, a discernible phenomenon of membrane rupture ensued, liberating the cellular contents into the surrounding milieu. Consequently, the treated bacterial cells exhibited an empty morphology, with the cytoplasm diffusing out of the ruptured membranes (Fig. 12b). This striking observation underscores the profound impact of ZnO on bacterial cell membranes, as it unequivocally demonstrates the nanoparticle's ability to induce membrane disruption. Notably, the TEM findings were found to be in alignment with the outcomes of various biochemical assays, including the quantification of reactive oxygen species (ROS), lipid peroxidation levels, and the release of cellular contents.

The physical contact between ZnO NPs and microbial cells can disrupt vital cellular processes, impede nutrient uptake, and induce cellular stress responses, further contributing to the antimicrobial effect of ZnO NPs.^88^ The antimicrobial activity of zinc oxide nanoparticles (ZnO NPs) involves direct interaction with bacterial cells, leading to the production of reactive oxygen species (ROS) in close proximity to the bacterial membrane.^88^ This mechanism, as suggested by previous studies, initiates oxidative damage to bacterial cells, beginning with the cell wall and progressing to the inner cytoplasmic membrane and peptidoglycan layer. This oxidative damage interferes with respiratory activities, causing slow leakage of RNA and proteins, as well as rapid leakage of K^+^ ions, ultimately resulting in bacterial death.^89^ Notably, the negatively charged nature of bacterial cells at physiological pH, attributed to the dissociation of carboxylic groups, contrasts with the positively charged properties of ZnO nanoparticles, which typically exhibit a zeta potential of +24 mV.^89^ This charge difference facilitates an interaction/electrostatic force between the negatively charged bacterial cells and positively charged ZnO, leading to the disruption of the cell wall and subsequent intracellular damage upon nanoparticle entry.^90^

Zhang *et al.* (2007) synthesized ZnO nanofluid using ultrasonication of commercially obtained agglomerated ZnO powder, which exhibited direct contact antibacterial activity against *E. coli* DH5α bacteria.^91^ The study revealed that increasing the concentration of ZnO NPs and reducing their particle size resulted in a higher bacterial death rate, indicating significant damage to the bacterial cell membrane post-treatment. Electrochemical measurements using a model dioleoyl-phosphatidylcholine monolayer confirmed the bacterial damage due to the interaction between bacterial cells and ZnO NPs. Interestingly, the presence of stabilizing agents such as polyethylene glycol and polyvinylpyrrolidone did not significantly affect the antibacterial activity of ZnO nanofluids.

Moreover, the intrinsic characteristics of ZnO NPs extend beyond direct interactions with microbial cells. With their elevated surface area and catalytic capabilities, these nanoparticles possess the capacity to engage in supplementary antimicrobial mechanisms. Through adherence to microbial surfaces, ZnO NPs facilitate their penetration into the cells, initiating antimicrobial actions by disrupting vital cellular functions and metabolic pathways. This multifaceted approach, combining direct cellular damage with interference at the molecular level, highlights the comprehensive antimicrobial efficacy of ZnO nanoparticles, making them invaluable in combating microbial infections and upholding hygiene standards across diverse applications.

### 4.2 Recent studies demonstrating the effectiveness of ZnO NPs against various pathogens

Recent studies have demonstrated the effectiveness of ZnO NPs against a wide range of pathogens, including bacteria, viruses, and fungi.^92^ For example, ZnO NPs have shown potent antibacterial activity against both Gram-positive and Gram-negative bacteria, including multidrug-resistant strains such as methicillin-resistant *Staphylococcus aureus* (MRSA) and carbapenem-resistant Enterobacteriaceae (CRE). Furthermore, ZnO NPs have exhibited antiviral activity against enveloped viruses like influenza virus and herpes simplex virus, as well as non-enveloped viruses such as norovirus and adenovirus.^93^ Additionally, ZnO NPs have demonstrated antifungal activity against various fungal pathogens, including *Candida species*.^94^

**Fig. 13:**
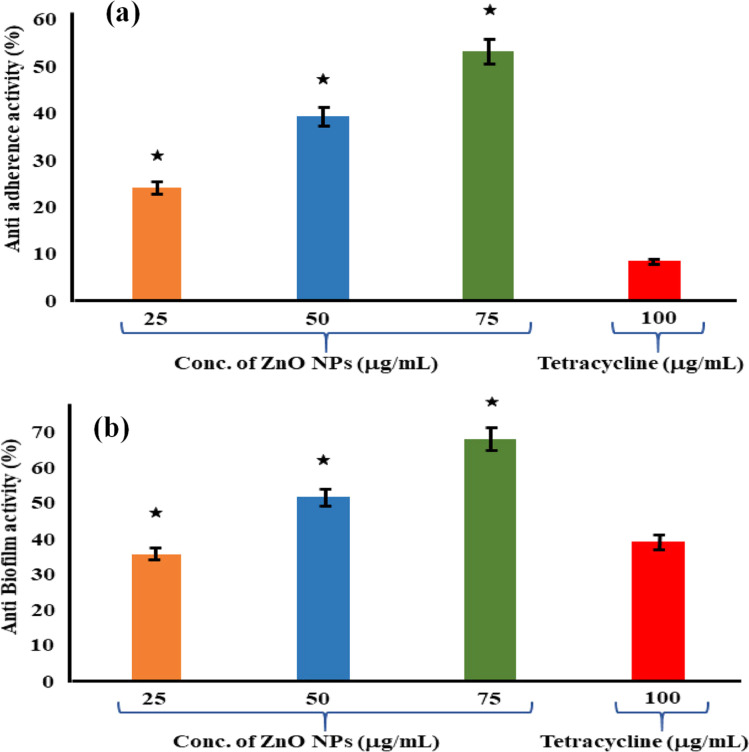
(a) Antiadherence assay and (b) antibiofilm assay using tetracycline as positive control and *S. aureus* as growth control. The experiment was evaluated based on triplicate results with standard deviation (*n* = 3, *p* < 0.05). * indicates a significant difference when compared to the negative control (NB only).^95^

**Fig. 14:**
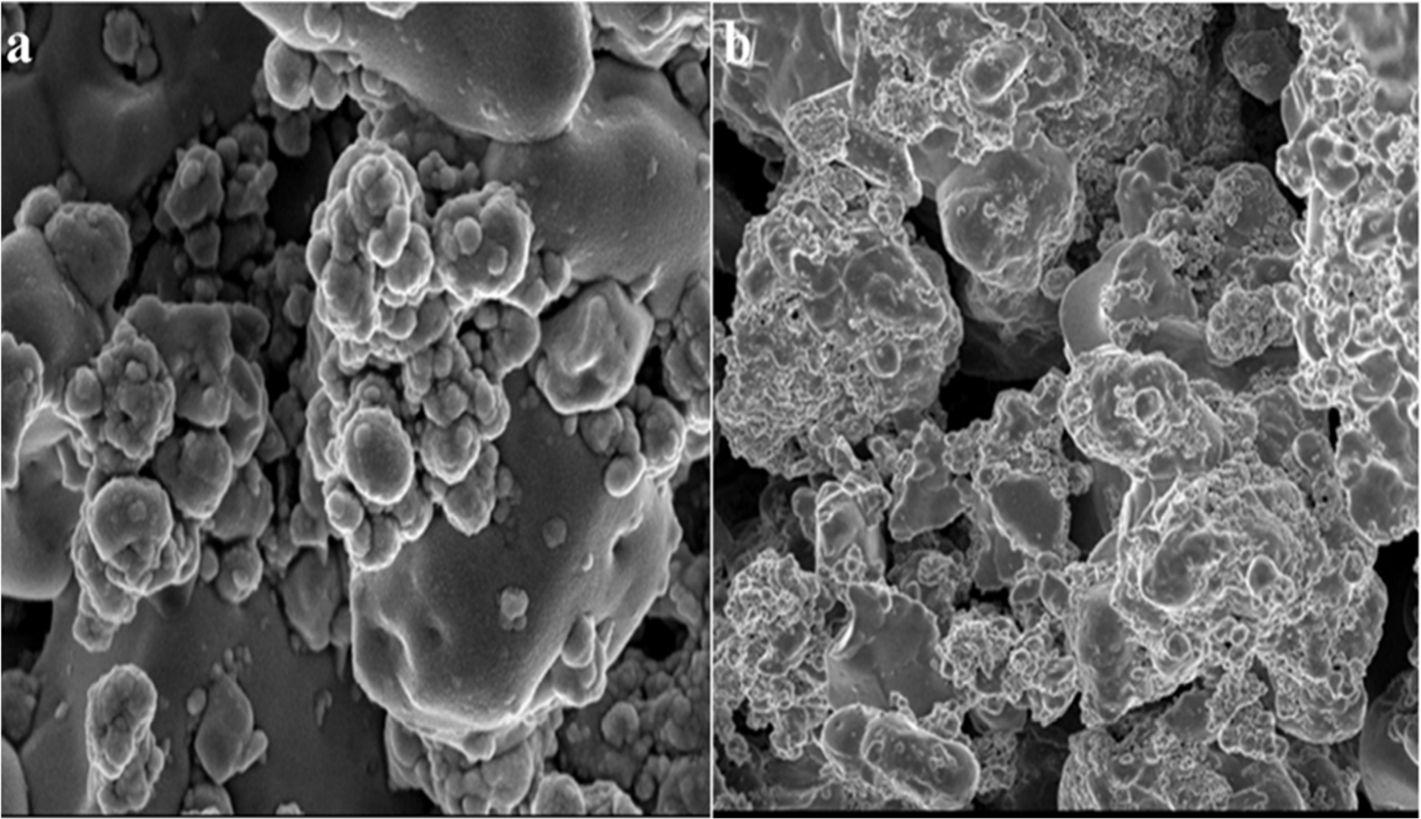
SEM micrographs of biofilm mass: (a) zinc oxide nanoparticles attached to the *S. aureus* biofilm, (b) biofilm of *S. aureus* disturbed after 24 h of treatment with ZnO NPs.^95^

Shobha *et al.* (2023) addressed the pressing issue of antimicrobial resistance by exploring the antibacterial potential of zinc oxide nanoparticles (ZnO NPs) synthesized using *Trichoderma asperellum* against two significant human pathogens, *Escherichia coli* and *Staphylococcus aureus*.^95^ The study demonstrated that biosynthesized ZnO NPs exhibited efficient antibacterial properties against both pathogens, as evidenced by inhibition zones ranging from 3 to 9 mm (Fig. 13a). Moreover, ZnO NPs effectively prevented biofilm formation and adherence of *Staphylococcus aureus*, a crucial aspect in combating infections caused by biofilm-forming bacteria. The minimum inhibitory concentration (MIC) of ZnO NPs showed effective antibacterial and antibiofilm actions against *Staphylococcus aureus* (Fig. 13b). Furthermore, ZnO NPs exhibited strong antiadherence activity, with significant reductions in bacterial biomass attachment observed as the concentration of ZnO NPs increased. Crystal violet staining of biofilms confirmed the inhibitory effect of ZnO NPs on biofilm development, with a marked reduction observed at 25 μg mL^−1^ concentration. The study also highlighted the superior antibiofilm activity of ZnO NPs compared to tetracycline, a conventional antibiotic. SEM micrographs revealed the mechanism of action of ZnO NPs against *Staphylococcus aureus*, indicating their adherence to the bacterial cell membrane and potential penetration, leading to cell death (Fig. 14). These findings underscore the potential of ZnO NPs as effective agents against drug-resistant *Staphylococcus aureus* infections, particularly in inhibiting biofilm formation, a critical factor in disease progression. Therefore, ZnO NPs hold promise as part of combination therapy for combating drug-resistant bacterial infections, especially those involving biofilm-associated pathogens like *Staphylococcus aureus*.

**Fig. 15:**
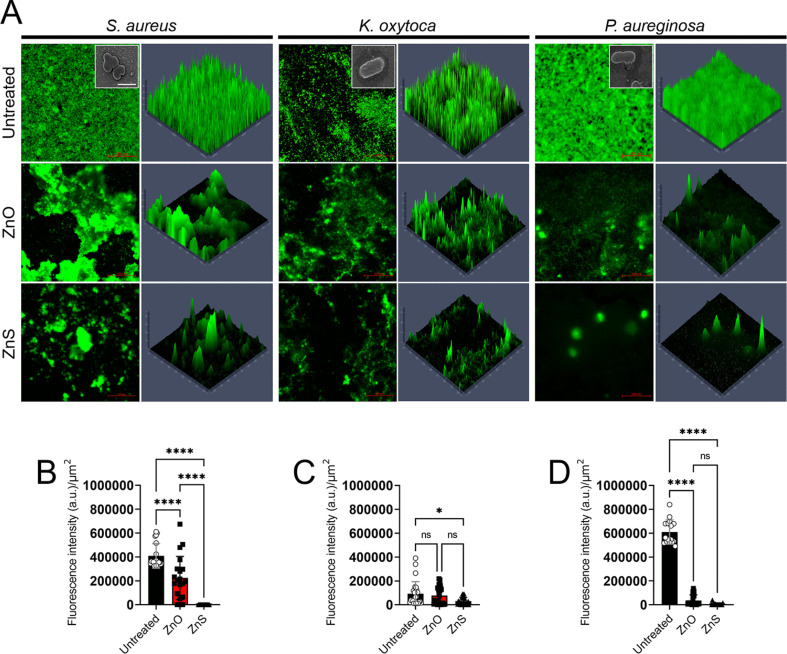
Confocal microscopy analysis of bacterial biofilms after 24 hours of growth in the presence of zinc oxide (ZnO) or zinc sulfide (ZnS) nanoparticles (NPs). Panel (A) displays representative images of untreated, ZnO-treated, or ZnS-treated biofilms, with bacterial biofilms grown alone or with varying concentrations of NPs. Green fluorescence (SYTO9) highlights bacteria, allowing observation of biofilm thickness and morphology through Z-stack reconstruction. Images captured at ×63 magnification with a scale bar of 200 μm. Insets show scanning electron microscopy (SEM) images of individual bacterial cells at ×100 magnification with a scale bar of 1 μm. Panels (B) to (D) quantify fluorescent intensity per square micrometer (μm^2^) for biofilms of *Staphylococcus aureus*, *Klebsiella oxytoca*, and *Pseudomonas aeruginosa*, respectively, grown with or without Zn NPs. Data analyzed using ImageJ within a 100 μm × 100 μm region of interest (ROI). Bars represent means, error bars show standard deviations (SDs), symbols denote individual ROIs (*n* = 20 for *S. aureus*; *n* = 25 for *K. oxytoca*; *n* = 16 for *P. aeruginosa*). Asterisks indicate *P* value significance (*, *P* < 0.05; ****, *P* < 0.0001), analyzed using ANOVA with Tukey's post hoc adjustment. “ns” denotes nonsignificant comparisons. Experiments repeated twice, yielding consistent results.^96^

In another study, Fulindi *et al.* (2023) investigated the bactericidal and antibiofilm activity of zinc oxide (ZnO) and zinc sulfide (ZnS) nanoparticles (NPs) against medically important bacteria, providing valuable insights for potential applications in medical and food preservation settings.^96^ The study aimed to analyze the efficacy of ZnS NPs against *Staphylococcus aureus*, *Klebsiella oxytoca*, and *Pseudomonas aeruginosa*, comparing it to the well-studied ZnO NPs. Using the XTT reduction assay to assess bacterial metabolic activity and the crystal violet assay to measure biofilm mass, the researchers found that both ZnS and ZnO NPs exhibited similar effectiveness in killing planktonic bacterial cells and reducing biofilm formation. Notably, *S. aureus* displayed higher susceptibility to both types of nanoparticles compared to *K. oxytoca* and *P. aeruginosa*. Confocal microscopy images (Fig. 15) revealed the inhibitory effect of Zn NPs on biofilm formation and their ability to cause architectural damage. The fluorescent intensity of biofilm images was quantified using ImageJ software, showing significant reductions in biofilm mass in the presence of Zn NPs. These findings highlight the potential of ZnS NPs as antibiofilm agents for various applications, including the treatment of wound infections and food preservation. Further studies are warranted to explore the full scope of their therapeutic benefits.

The studies by Yassin *et al.* (2023),^97^ Urge *et al.* (2023),^98^ Almaary *et al.* (2023),^99^ Jawad (2023),^100^ and Motelică *et al.* (2023)^101^ collectively provide valuable insights into the antibacterial potential of zinc oxide nanoparticles (ZnO NPs), each offering unique perspectives on synthesis methods, characterization techniques, and antibacterial efficacy.

Yassin *et al.* (2023) focused on the anticandidal activity of green-synthesized ZnO-NPs using *Camellia sinensis* leaf extract.^97^ Their findings highlighted significant inhibition of candidal pathogens, particularly *Candida tropicalis* and *Candida glabrata* strains. Moreover, synergistic effects with conventional antifungal agents demonstrated the potential of ZnO-NPs as adjunct therapies for candidal infections.

Similarly, Urge *et al.* (2023) explored the antibacterial properties of ZnO NPs synthesized using garlic bulb and ginger extracts.^98^ Their results demonstrated potent inhibition of both Gram-negative and Gram-positive bacteria, with the mixture of garlic bulb and ginger extracts yielding ZnO NPs with enhanced antibacterial activity. This suggests the potential synergistic effects of combining plant extracts in nanoparticle synthesis for improved antibacterial efficacy. Almaary *et al.* (2023) investigated the antibacterial effectiveness of bioprepared ZnO-NPs synthesized using the flower extract of Hibiscus sabdariffa.^99^ Their study revealed significant antibacterial activity against nosocomial bacterial pathogens, highlighting the potential of ZnO-NPs as therapeutic agents for combating antibacterial resistance. Additionally, synergistic effects with fosfomycin further underscored the clinical relevance of ZnO-NPs as combination therapies for nosocomial infections.

In contrast, Jawad (2023) synthesized ZnO NPs *via* laser ablation and evaluated their antibacterial activity against *Proteus mirabilis* isolates.^100^ The results indicated strong antibacterial and antibiofilm activity of ZnO NPs, surpassing the efficacy of conventional antibiotics. Moreover, the study suggested the potential application of ZnO NPs as preservatives for medical devices, such as urinary catheters, by preventing microbial biofilm formation.

Motelică *et al.* (2023) contributed to the understanding of ZnO NPs synthesis *via* forced solvolysis and their antibacterial activity against a range of bacterial strains.^101^ Their findings demonstrated robust inhibition of bacterial growth, particularly in water purification applications, highlighting the versatility of ZnO NPs as antibacterial agents in various environmental settings.

Overall, these studies collectively underscore the significant antibacterial potential of ZnO nanoparticles synthesized through green and solvolysis methods. The diverse approaches employed in nanoparticle synthesis and characterization contribute to our comprehensive understanding of their antibacterial efficacy and pave the way for the development of novel antibacterial agents with broad-spectrum activity and reduced environmental impact.

Khan *et al.* (2023)^102^ and Singh *et al.* (2023) presented compelling evidence of the antibacterial efficacy of zinc oxide nanoparticles (ZnO-NPs) synthesized through different methods against multidrug-resistant (MDR) bacterial pathogens. Khan *et al.* (2023) focused on the synthesis of ZnO-NPs using *Cassia siamea* leaf extract, demonstrating their ability to suppress quorum-mediated virulence factors and inhibit biofilm formation in clinical MDR isolates of *Pseudomonas aeruginosa* and *Chromobacterium violaceum*.^102^ The study highlighted the significant reduction in virulence factors and biofilm formation, emphasizing the potential of ZnO@Cs-NPs as alternative therapeutic agents for managing pathogenic infections. The findings also indicated strong antibacterial efficacy by showing disruption of membrane permeability in bacterial cells, consistent with the study by Yan *et al.* (2021).^103^

In contrast, Singh *et al.* (2023) synthesized ZnO nanoflakes using a co-precipitation method and evaluated their antibacterial activity against carbapenem-resistant (CR) clinical isolates.^34^ The study demonstrated the inhibitory action of ZnO nanoflakes against various MDR bacterial strains, including *Escherichia coli*, *Klebsiella pneumoniae*, *Acinetobacter baumannii*, and *Pseudomonas aeruginosa*. Additionally, the combination of ZnO nanoflakes with Meropenem exhibited synergistic activity against CR pathogens, suggesting the potential of ZnO nanoflakes as nano-antibiotics for treating infections caused by carbapenem-resistant Enterobacteriaceae (CRE) bacteria.

Comparatively, both studies underscore the significant antibacterial potential of ZnO-based nanomaterials against MDR bacterial pathogens. While Khan *et al.*^102^ focused on the suppression of virulence factors and biofilm formation, Singh *et al.*^104^ investigated the inhibitory action of ZnO nanoflakes against CR clinical isolates and demonstrated synergistic activity with conventional antibiotics. The complementary findings from these studies highlight the versatility and promising therapeutic applications of ZnO nanoparticles in combating antibiotic resistance and managing infectious diseases. Overall, the studies provide valuable insights into the development of novel nano-antibiotics for addressing the global challenge of antibiotic-resistant infections.

**Fig. 16:**
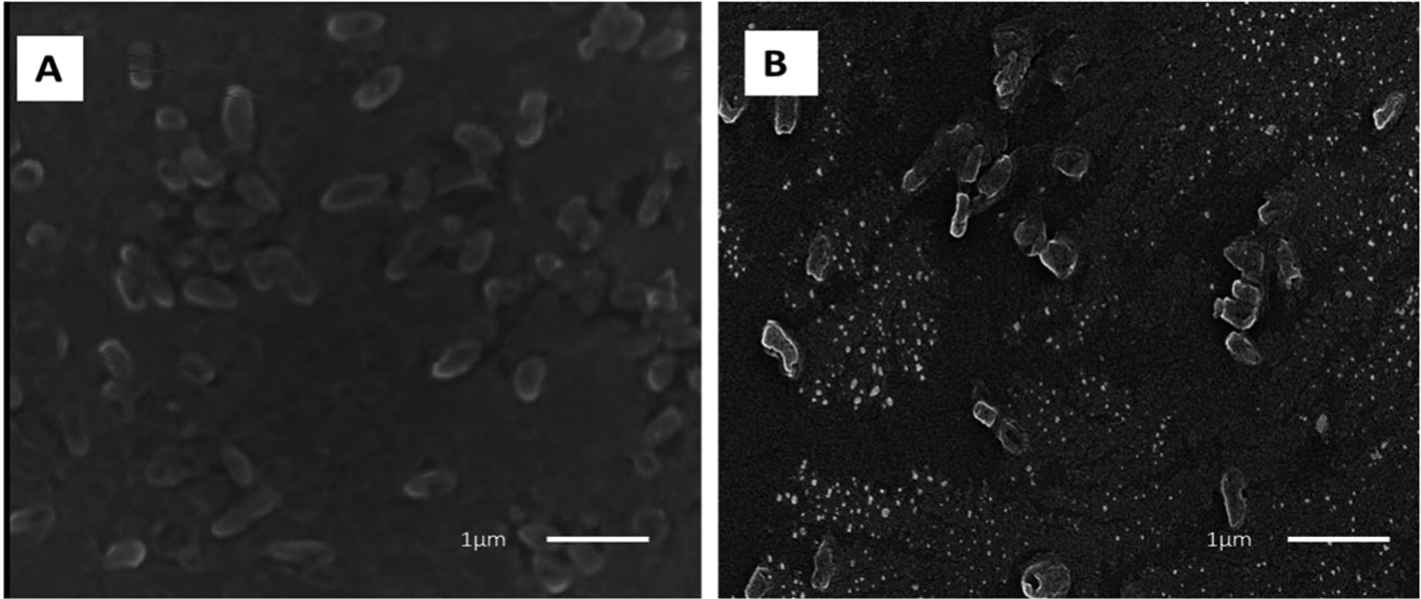
Scanning electron micrograph of *P. aeruginosa* isolates: (A) before and (B) after treatment with ZnO-NPs.^105^

Alotaibi *et al.* (2022) addressed the escalating challenge of bacterial resistance by advocating for the development of novel antibacterial agents. In this context, metal nanoparticles emerge as promising candidates to counter antibiotic resistance.^105^ The study employs *Gardenia thailandica* methanol extract (GTME) to biogenically synthesize zinc oxide nanoparticles (ZnO-NPs). Comprehensive characterization of ZnO-NPs is conducted using various analytical techniques including UV spectroscopy, FTIR, scanning and transmission electron microscopes, dynamic light scattering, and X-ray diffraction. Evaluation of the antibacterial activity of ZnO-NPs is performed both *in vitro* and *in vivo* against *Pseudomonas aeruginosa* clinical isolates. The minimum inhibitory concentration values of ZnO-NPs range from 2 to 64 μg mL^−1^, significantly affecting membrane integrity and enhancing inner and outer membrane permeability. Scanning electron microscope examination reveals that ZnO-NPs induce morphological distortions and deformities in treated *P. aeruginosa* cells (Fig. 16). *In vivo* studies, including biochemical parameters and histological investigations, demonstrate the protective effect of ZnO-NPs against the harmful effects of *P. aeruginosa* on lung, liver, and kidney tissues. Additionally, LC-ESI-MS/MS analysis tentatively identifies 57 phytochemical compounds for the first time. The study suggests that GTME represents a valuable resource for the synthesis of ZnO-NPs with promising antibacterial activity.

Krishnamoorthy *et al.* (2022) and Hayat *et al.* (2022)^106^ contribute significant insights into the antibacterial potential of zinc oxide nanoparticles (ZnO NPs) against multidrug-resistant (MDR) Gram-negative foodborne pathogens.^107^ Both studies highlight the urgency of addressing the escalating challenge of antibiotic resistance in the food industry and propose nanotechnology-based approaches as promising solutions.

Krishnamoorthy *et al.* (2022) investigate the antibacterial activity of ZnO NPs against β-lactam-resistant Gram-negative food pathogens, including *Escherichia coli*, *Pseudomonas aeruginosa*, *Salmonella typhi*, *Serratia marcescens*, *Klebsiella pneumoniae*, and *Proteus mirabilis*.^107^ Their findings demonstrate the broad-spectrum action of ZnO NPs, with minimal inhibitory concentrations ranging from 0.04 to 0.08 mg mL^−1^. The mechanism of action involves the elevation of reactive oxygen species (ROS) and malondialdehyde levels, leading to membrane lipid peroxidation and subsequent membrane damage, protein denaturation, DNA damage, and ultimately cell death. These results underscore the potential of ZnO NPs as potent antibacterial agents against β-lactam-resistant Gram-negative pathogens, offering a promising avenue for combating antibiotic resistance in the food industry.

On the other hand, Hayat *et al.* (2022) focus on the green synthesis of ZnO NPs using the aqueous leaf extract of *Acacia arabica* and evaluate their antibacterial efficacy against foodborne pathogens.^106^ Their comprehensive study includes characterization of the synthesized nanoparticles, antibacterial susceptibility testing using agar well diffusion and broth microdilution assays, assessment of biofilm formation and exopolysaccharide (EPS) production inhibition, antioxidant potential evaluation, and cytotoxicity studies. The results reveal significant antibacterial activity of green-synthesized ZnO NPs against foodborne pathogens, with diameter zones of inhibition ranging from 16 to 30 nm and MIC/MBC values ranging from 31.25 to 62.5 μg mL^−1^. Moreover, the nanoparticles exhibit bactericidal potential, inhibit biofilm formation and EPS production, demonstrate antioxidant activity, and show non-toxicity to HeLa cell lines. These findings highlight the potential of green-synthesized ZnO NPs as safe and effective alternatives to chemical drugs for combating antibiotic-resistant foodborne pathogens.

In summary, both studies provide valuable insights into the application of ZnO NPs as antibacterial agents against MDR Gram-negative foodborne pathogens. While Krishnamoorthy *et al.* (2022) elucidate the mechanism of action of ZnO NPs, Hayat *et al.* (2022) emphasized the green synthesis approach and comprehensive evaluation of antibacterial efficacy, biofilm inhibition, antioxidant activity, and cytotoxicity.^106^ Together, these studies underscore the potential of ZnO NPs as versatile and effective nanomaterials for addressing antibiotic resistance in the food industry.

**Fig. 17:**
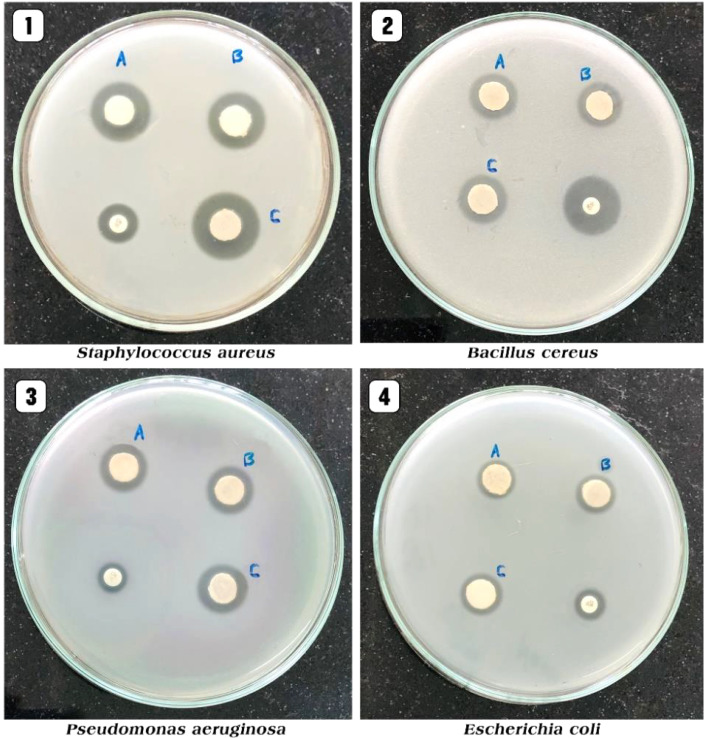
The antibacterial effects, measured by the zone of inhibition (mm), of different concentrations of ZnO NPs. Panels 1, 2, 3, 4 represent concentrations of 10 μg mL^−1^, 20 μg mL^−1^, 30 μg mL^−1^, and the standard, respectively, against various pathogens.^108^

The study of Abdelbaky *et al.* (2022) explored the antibacterial potency of zinc oxide nanoparticles (ZnO NPs) synthesized using an aqueous leaf extract of *Pelargonium odoratissimum* (L.).^108^ The study employed various characterization techniques to confirm the synthesis and properties of the ZnO NPs. The antibacterial activity of these nanoparticles was evaluated against four foodborne pathogenic bacterial strains using the disk diffusion assay. The results indicated significant antibacterial effects, with *Staphylococcus aureus* exhibiting the highest sensitivity. Additionally, the ZnO NPs showed superior antibacterial activity compared to both the positive control gentamycin and the aqueous leaf extract of *P. odoratissimum*. Furthermore, the study demonstrated that the antibacterial activity of the biosynthesized ZnO NPs was more pronounced against Gram-positive bacteria (GPB) compared to Gram-negative bacteria (GNB). This trend is consistent with previous findings and may be attributed to differences in cell wall structure between GPB and GNB (Fig. 17). The study also highlighted the anti-inflammatory properties of the ZnO NPs, suggesting their potential application in biomedical and pharmaceutical industries as safe alternatives to synthetic substances for antibacterial and anti-inflammatory purposes.

The studies by Husain *et al.* (2022),^109^ El-Masry *et al.* (2022),^110^ Álvarez-Chimal *et al.* (2021),^111^ Ali *et al.* (2021),^112^ Du *et al.* (2021),^113^ Fadwa *et al.* (2021)^114^, and Abdelraheem *et al.* (2021)^115^ collectively shed light on the potential of zinc oxide nanoparticles (ZnO-NPs) as effective antimicrobial agents against various multidrug-resistant (MDR) bacterial strains.

Husain *et al.* (2022)^109^ and El-Masry *et al.* (2022)^110^ focus on the antibiofilm and antibacterial properties of ZnO-NPs against Gram-positive and Gram-negative bacteria. Husain *et al.* (2022) synthesized ZnO-NPs using extract of *Plumbago zeylanica* and demonstrated their ability to inhibit and eradicate biofilms formed by *E. coli*, *S. aureus*, and *P. Aeruginosa*.^109^ Similarly, El-Masry *et al.* (2022) investigated the antibacterial properties of ZnO NPs against enterotoxigenic *S. aureus*, showcasing significant inhibition of bacterial growth and reduction of enterotoxin A concentration.^110^

Álvarez-Chimal *et al.* (2021) further explore the antibacterial properties of ZnO-NPs synthesized using the Mexican plant *Dysphania ambrosioides*.^111^ Their study reveals the sensitivity of various bacterial strains, including *S. aureus*, *S. epidermidis*, *E. coli*, *P. aeruginosa*, *Aggregatibacter actinomycetemcomitans*, *Porphyromonas gingivalis*, *Prevotella intermedia*, *Streptococcus mutans*, and *Streptococcus sanguinis*, to both synthesized and commercial ZnO-NPs.

Ali *et al.* (2021)^112^ and Du *et al.* (2021)^113^ delve into the mechanisms underlying the antibacterial activity of ZnO-NPs. Ali *et al.* (2021)^112^ synthesized ZnO-NPs using seed extract of *Butea monosperma* and demonstrated their ability to disrupt quorum sensing mechanisms in *P. aeruginosa*, leading to inhibition of virulence factors and biofilm formation. On the other hand, Du *et al.* (2021)^113^ investigated the visible-light-driven photocatalytic inactivation mechanism of amino-functionalized hydrophilic ZnO NPs against *S. aureus*, elucidating the role of electron transfer in antibacterial activity. Fadwa *et al.* (2021)^114^ and Abdelraheem *et al.* (2021) focus on the potential of ZnO-NPs in combination with antibiotics to combat MDR bacterial strains.^115^ Fadwa *et al.* (2021) evaluated the MIC and fractional inhibitory concentration index (FICI) of ZnO-NPs in combination with colistin, ciprofloxacin, and meropenem against *E. coli* and *A. baumannii*, highlighting their synergistic effects.^114^ Abdelraheem *et al.* (2021) investigate the antibiofilm and antimicrobial effects of ZnO-NPs against methicillin-resistant *S. aureus* (MRSA), vancomycin-resistant *S. aureus* (VRSA), and linezolid-resistant *S. aureus* (LRSA), emphasizing the potential of ZnO-NPs to reduce biofilm formation and drug resistance in *S. aureus* isolates.^115^

Collectively, these studies underscore the versatile antimicrobial properties of ZnO-NPs and their potential applications in combating MDR bacterial strains, offering promising avenues for the development of novel antibacterial agents and therapies.

Table 2 provides a comprehensive overview of various studies investigating the antibacterial and antibiofilm effects of zinc oxide nanoparticles (ZnO NPs) against different bacterial pathogens. The table summarizes key details, including the authors, types of bacterial pathogens studied, and the notable effects of ZnO NPs on these pathogens. The studies encompass a wide range of bacterial strains, both Gram-positive and Gram-negative, highlighting the broad-spectrum antibacterial activity of ZnO NPs. The findings consistently demonstrate the potential of ZnO NPs to inhibit bacterial growth, disrupt biofilm formation, and enhance the efficacy of conventional antibiotics, suggesting their promising application in combating antibiotic-resistant infections.

**Table 2:** Summary of studies on the antibacterial and antibiofilm effects of zinc oxide nanoparticles (ZnO NPs) against various pathogens

Author	Bacterial types	Summary of effects on pathogens
Shobha *et al.*^95^	*Escherichia coli*	Efficient antibacterial properties against both pathogens, inhibition of biofilm formation
	*Staphylococcus aureus*	
Fulindi *et al.*^8^	*Staphylococcus aureus*	Effective against multiple bacterial strains, inhibition of biofilm formation
	*Klebsiella oxytoca*, *Pseudomonas aeruginosa*	
Yassin *et al.*^97^	*Candida tropicalis*	Significant inhibition of candidal pathogens, potential synergistic effects with antifungal agents
	*Candida glabrata*	
Urge *et al.*^98^	Gram-negative and Gram-positive bacteria	Potent inhibition of bacterial growth, enhanced antibacterial activity with combined plant extracts
Almaary *et al.*^99^	Nosocomial bacterial pathogens	Significant antibacterial activity against nosocomial bacterial pathogens, synergistic effects with fosfomycin
Jawad^100^	*Proteus mirabilis* isolates	Strong antibacterial and antibiofilm activity against specific bacterial strains, potential application in medical device preservation
Motelică *et al.*^101^	Gram-negative strains (*Salmonella enterica* serovar Typhimurium, *Pseudomonas aeruginosa*, and *Escherichia coli*), and Gram-positive strains (*Enterococcus faecalis*, *Bacillus subtilis*, *Staphylococcus aureus*, and *Bacillus cereus*)	Robust inhibition of bacterial growth, particularly in water purification applications
Khan *et al.*^102^	Clinical multidrug-resistant isolates	Suppression of quorum-mediated virulence factors and biofilm formation in clinical MDR isolates, potential as alternative therapeutic agents
Singh *et al.*^34^	Carbapenem-resistant clinical isolates	Inhibitory action against carbapenem-resistant clinical isolates, synergistic activity with conventional antibiotics
Alotaibi *et al.*^105^	*Pseudomonas aeruginosa* clinical isolates	Significant antibacterial activity against specific clinical isolates, potential for therapeutic applications
Krishnamoorthy *et al.*^107^	β-Lactam-resistant Gram-negative food pathogens	Broad-spectrum antibacterial activity against specific foodborne pathogens
Hayat *et al.*^106^	Foodborne pathogens	Significant antibacterial activity against foodborne pathogens, inhibition of biofilm formation and EPS production
Abdelbaky *et al.*^108^	Foodborne pathogenic bacterial strains	Significant antibacterial potency against specific foodborne pathogenic strains, superior activity against Gram-positive bacteria
Husain *et al.*^109^	*E. coli*, *S. aureus*, *P. aeruginosa*	Inhibition and eradication of biofilms formed by specific bacterial strains
El-Masry *et al.*^110^	Enterotoxigenic *S. aureus*	Antibacterial properties against enterotoxigenic *S. aureus*, reduction of enterotoxin A concentration
Álvarez-Chimal *et al.*^111^	Various bacterial strains	Sensitivity of various bacterial strains to ZnO-NPs, including specific pathogens
Ali *et al.*^112^	*P. aeruginosa*	Disruption of quorum sensing mechanisms in *P. aeruginosa*, inhibition of virulence factors and biofilm formation
Du *et al.*^113^	*S. aureus*	Visible-light-driven photocatalytic inactivation mechanism against *S. aureus*, elucidation of electron transfer role
Fadwa *et al.*^114^	*E. coli*, *A. baumannii*	Synergistic effects with antibiotics against specific bacterial strains, reduction of biofilm formation and drug resistance
Abdelraheem *et al.*^115^	Methicillin, vancomycin and linezolid resistant	Antibiofilm and antimicrobial effects against specific methicillin-resistant *S. aureus* strains
	*S. aureus* (MRSA, VRSA and LRSA)	

### 4.3 Impacts of different conditions on antibacterial efficacy

Several factors contribute to the effectiveness of ZnO nanoparticles (NPs) as antimicrobial agents, including their size, shape, and surface characteristics.^116^ Typically, smaller nanoparticles exhibit heightened antimicrobial activity due to their larger surface area-to-volume ratio, facilitating increased reactive oxygen species (ROS) generation and cellular uptake.^117^ Moreover, the shape of ZnO NPs plays a significant role, with certain shapes such as nanorods and nanospheres demonstrating superior antimicrobial efficacy compared to others. Additionally, surface modifications, such as altering surface charge and functionalization, can enhance the interaction between ZnO NPs and microbial cells, thereby augmenting their antimicrobial activity.^118,119^

ZnO nanoparticles exhibit potent antimicrobial properties mediated by mechanisms involving ROS generation and membrane disruption. Recent studies have underscored their efficacy against a wide range of pathogens, and their antimicrobial effectiveness can be further enhanced by optimizing their size, shape, and surface properties.^120,121^ These findings highlight the versatility of ZnO NPs as promising antimicrobial agents for various healthcare applications.

To enhance the antibacterial activity of metal oxide nanomaterials, it is crucial to optimize various parameters, including the size of the nanomaterials, concentration, temperature, capping agent, and reducing agent.^122^ Metal oxides possess a large surface area and high surface energy, which can lead to agglomeration and decrease their antibacterial activity. Stabilizing agents, such as polymers and plant extracts, are often employed to prevent agglomeration and enhance antibacterial efficacy.^123^

Studies have investigated the effect of ZnO particle size on antibacterial activity against different pathogens.^124,125^ Decreasing particle size has been associated with increased antibacterial activity. Factors such as pH and annealing temperature during synthesis also influence ZnO particle size and antibacterial efficacy.^124^ Higher pH levels and lower annealing temperatures have been correlated with enhanced antibacterial activity due to increased ROS generation.^124^ For example, Babayevska *et al.* (2022) aimed to synthesize zinc oxide (ZnO) nano- and microparticles and investigate how variations in shape and size influence cytotoxicity towards both normal and cancer cells, as well as antibacterial activity against two distinct bacterial strains.^125^ The researchers employed facile chemical and physical methods to fabricate ZnO nano- and microparticles, followed by a meticulous characterization of their crystal structure, morphology, textural properties, and photoluminescent properties. This characterization encompassed techniques such as powder X-ray diffraction, electron microscopy, nitrogen adsorption/desorption measurements, and photoluminescence spectroscopy.

Their findings revealed that the ZnO structures obtained were highly crystalline and monodispersed, exhibiting an intense green emission. Notably, ZnO nanoparticles (NPs) and nanorods (NRs) exhibited superior antibacterial activity against *Escherichia coli* and *Staphylococcus aureus* compared to microparticles, owing to their larger specific surface area. However, ZnO hierarchical structures (HSs) also demonstrated significant inhibition of bacterial growth, particularly at higher concentrations. Interestingly, *S. aureus* proved to be more susceptible to ZnO particles than *E. coli.*

Furthermore, the study shed light on the differential cytotoxicity of ZnO nano- and microparticles towards cancer cell lines and normal cells. ZnO NPs and NRs exhibited greater harm to cancer cells compared to normal cells when tested at equivalent concentrations. These findings underscore the potential of ZnO nanostructures as promising candidates for both antibacterial and anticancer applications, while emphasizing the importance of understanding their size and shape-dependent effects on biological systems.

In a different study conducted by Padmavathy and Vijayaraghavan (2008), the effect of particle size on the antimicrobial activity of zinc oxide (ZnO) nanoparticles was investigated.^126^ ZnO nanoparticles were synthesized using two different methods: base hydrolysis of zinc acetate in a 2-propanol medium and precipitation method using zinc nitrate and sodium hydroxide. The synthesized nanoparticles were characterized using various techniques such as X-ray diffraction (XRD) analysis, transmission electron microscopy (TEM), and photoluminescence (PL) spectroscopy.

The bacteriological tests, including minimum inhibitory concentration (MIC) and disk diffusion assays, were performed using different concentrations of ZnO nanoparticles in both solid agar plates and liquid broth systems, with Luria–Bertani and nutrient agar media employed. The results from the bacteriological study revealed an enhanced biocidal activity of ZnO nanoparticles compared to bulk ZnO in multiple experiments. Notably, the study demonstrated that the bactericidal efficacy of ZnO nanoparticles increased with decreasing particle size.

The observed increase in antimicrobial activity with decreasing particle size suggests that smaller ZnO nanoparticles possess greater efficacy against bacteria. This phenomenon could be attributed to several factors. Firstly, the increased surface area-to-volume ratio of smaller nanoparticles provides more active sites for interaction with bacterial cells, facilitating enhanced bactericidal activity. Additionally, smaller nanoparticles may exhibit higher surface reactivity and greater surface energy, leading to enhanced interactions with bacterial cell membranes.

Furthermore, the abrasiveness and surface oxygen species of ZnO nanoparticles likely contribute to their biocidal properties. The abrasive nature of ZnO nanoparticles can disrupt bacterial cell membranes, leading to cell lysis and death. Moreover, the presence of surface oxygen species, such as hydroxyl radicals, can induce oxidative stress in bacterial cells, resulting in damage to cellular components and ultimately cell death.

Overall, the findings from this study suggest that the antimicrobial activity of ZnO nanoparticles is influenced by their particle size, with smaller nanoparticles exhibiting enhanced biocidal efficacy. The proposed mechanisms underlying this phenomenon involve both the physical properties (abrasiveness) and chemical properties (surface oxygen species) of ZnO nanoparticles, highlighting their potential as effective antimicrobial agents.

The studies conducted by Stanković *et al.* (2013)^127^ and Silva *et al.* (2019)^128^ collectively provide insights into the effect of zinc oxide (ZnO) nanoparticle size on their antibacterial potency. Stanković *et al.* (2013) synthesized ZnO particles of different sizes and morphologies using various surface stabilizing agents through a low-temperature hydrothermal procedure.^127^ They found that ZnO particles consisting of nanospherical particles exhibited the highest microbial cell reduction rate, indicating enhanced antibacterial activity. Moreover, all examined ZnO samples demonstrated significant bacteriostatic activity, suggesting that ZnO nanoparticles possess inherent antibacterial properties regardless of their size. Silva *et al.* (2019) investigated the influence of ZnO nanoparticle size and surface modification on antibacterial activity against *S. aureus* and *E. coli*.^128^ They found that smaller ZnO nanoparticles exhibited higher antibacterial activity, with smaller nanoparticles showing bactericidal activity and larger nanoparticles demonstrating bacteriostatic activity. Additionally, the use of a surface modifier led to an increase in the minimum inhibitory concentration (MIC) and minimum bactericidal concentration (MBC), indicating a reduction in antibacterial potency. This study underscores the significance of nanoparticle size and surface modification in determining antibacterial efficacy.

Overall, these studies collectively suggest that the size of ZnO nanoparticles plays a crucial role in determining their antibacterial potency. Smaller nanoparticles generally exhibit higher antibacterial activity, potentially due to their larger surface area-to-volume ratio, which facilitates greater interaction with bacterial cells. Additionally, surface modifications and stabilizing agents may influence the antibacterial efficacy of ZnO nanoparticles. Therefore, optimizing nanoparticle size and surface properties is essential for enhancing their antibacterial activity and expanding their applications in healthcare.

In the pursuit of enhancing the antibacterial efficacy of zinc oxide nanoparticles (ZnO NPs), researchers have explored various synthesis methods to achieve precise control over their morphologies and sizes. Among these methods, green synthetic approaches have garnered considerable attention and emerged as prominent techniques for several compelling reasons.

A noteworthy study by Ogunyemi *et al.* (2019) exemplifies the potential of green synthesis for producing ZnO NPs using plant extracts, offering a promising alternative to conventional chemical methods.^129^ In their work, ZnO NPs were synthesized utilizing extracts from chamomile flower (*Matricaria chamomilla* L.), olive leaves (*Olea europaea*), and red tomato fruit (*Lycopersicon esculentum* M.). Through a comprehensive characterization process involving UV-visible spectroscopy, Fourier transform infrared spectroscopy (FTIR), X-ray diffraction (XRD), transmission electron microscopy (TEM), and scanning electron microscopy (SEM) with energy-dispersive X-ray spectroscopy (EDS) profiling, the synthesized ZnO NPs were thoroughly analyzed. The XRD studies confirmed the presence of pure crystalline ZnO nanoparticles, with each plant extract yielding distinct size distributions. Notably, ZnO NPs synthesized using *Olea europaea* exhibited the smallest size range, ranging from 40.5 to 124.0 nm, as observed through SEM. This finding was corroborated by TEM analysis, which revealed an average particle size of 48.2 nm, consistent with XRD data. Furthermore, the antibacterial effects of ZnO NPs synthesized using *Olea europaea* extract were investigated against *Xanthomonas oryzae* pv. *oryzae* (Xoo) strain GZ 0003, a bacterium responsible for bacterial leaf blight diseases in rice.

The results indicated a significant inhibition zone of 2.2 cm at 16.0 μg mL^−1^, highlighting the efficacy of ZnO NPs synthesized with *Olea europaea* extract compared to those synthesized with other plant extracts. Moreover, the study elucidated the impact of ZnO NPs on various bacterial activities, including growth inhibition, biofilm formation, swimming motility, and disruption of bacterial cell membranes, underscoring the potential of ZnO NPs as biocontrol agents against bacterial leaf blight diseases in rice.

Overall, the study by Ogunyemi *et al.*^129^ underscores the promise of green synthesis approaches for fabricating ZnO NPs with tailored properties and potent antibacterial activity, offering novel solutions for combating bacterial infections and agricultural disease.

Furthermore, the integration of zinc oxide nanoparticles (ZnO NPs) into polymer matrices *via* techniques like electrospinning has emerged as a promising strategy to enhance their antibacterial properties for specific applications, notably wound dressing.^130^ Electrospinning facilitates the fabrication of microfibers characterized by high surface area and porosity, making them an ideal platform for incorporating ZnO NPs. By dispersing ZnO NPs within polymer solutions prior to electrospinning, homogeneous distribution throughout the microfibers can be achieved, thereby enhancing the antimicrobial activity of the resulting composite materials.^131,132^ Moreover, the fibrous structure of the microfibers promotes efficient wound exudate management and tissue regeneration while preventing microbial colonization, making them suitable for advanced wound dressing applications.^133^ For example, Shalumon *et al.* (2011) investigated the preparation of sodium alginate (SA)/poly (vinyl alcohol) (PVA) fibrous mats *via* electrospinning technique, incorporating ZnO nanoparticles synthesized with different concentrations into the composite nanofibers.^134^ Characterization studies confirmed the successful incorporation of ZnO nanoparticles, with the composite mats demonstrating antibacterial activity against *Staphylococcus aureus* and *Escherichia coli*. The study highlighted the potential of these composite mats for wound dressing applications, pending identification of the optimal ZnO concentration for maximal antibacterial activity with minimal cytotoxicity.

Similarly, Alavi *et al.* (2020) emphasized the importance of controllable release of Zn^2+^ ions from ZnO nanoparticles in physiological media to achieve appropriate antibacterial effects.^135^ They discussed the potential of coupling ZnO nanoparticles with natural polymers like cellulose, chitosan, and alginate to create nanocomposites with enhanced mechanical and antibacterial properties for wound healing applications. Rodríguez *et al.* (2014) evaluated nanocomposite mats based on poly(d,l-lactide) nanofibers incorporating different concentrations of zinc oxide nanoparticles, fabricated using electrospinning and electrospraying techniques.^136^ The presence of ZnO nanoparticles improved the mechanical properties of the mats and exhibited antibacterial activity against *Escherichia coli* and *Staphylococcus aureus*, demonstrating their potential as antimicrobial wound dressings.

Furthermore, Abdalkarim *et al.* (2017) explored the fabrication of bioactive nanofibrous membranes using cellulose nanocrystal-ZnO nanohybrids as reinforcing materials in biodegradable poly(3-hydroxybutyrate-*co*-3-hydroxy-valerate) (PHBV) *via* electrospinning.^137^ The resulting nanofibrous membranes exhibited improved mechanical properties, thermal stability, and antibacterial activity against *Escherichia coli* and *Staphylococcus aureus*, showcasing their potential as antibacterial wound dressings.

The aforementioned studies collectively highlight the effectiveness of integrating ZnO NPs into polymer matrices *via* electrospinning to enhance their antibacterial properties, paving the way for advanced wound dressing materials with improved therapeutic efficacy and biocompatibility.

## 5 UV protective properties of zinc oxide nanoparticles

Zinc oxide nanoparticles (ZnO NPs) are increasingly recognized for their remarkable ability to provide robust UV protection, which arises from a sophisticated interplay of mechanisms operating at the nanoscale.^137^ At the forefront of these mechanisms lies the exceptional optical properties inherent to ZnO NPs.^137^ With their wide bandgap, ZnO NPs possess the capacity to efficiently absorb and scatter UV radiation, thereby acting as a formidable shield against harmful ultraviolet rays.^138^ This inherent property allows ZnO NPs to form a physical barrier that intercepts UV radiation, preventing its penetration into underlying materials or skin tissues.

A noteworthy attribute contributing to the efficacy of ZnO NPs in UV protection is their impressive photostability. Even under prolonged exposure to sunlight or artificial UV sources, ZnO NPs retain their UV-protective properties without undergoing degradation or loss of effectiveness.^139^ This enduring photostability ensures consistent and reliable UV shielding over extended periods, making ZnO NPs a preferred choice for applications demanding sustained protection against UV radiation.

Moreover, beyond their role as UV blockers, ZnO NPs exhibit intrinsic antimicrobial properties that further augment their effectiveness in UV protection. By impeding the growth and proliferation of microorganisms on surfaces or skin subjected to UV exposure, ZnO NPs contribute to the preservation of the protective barrier, thereby enhancing overall UV protection.^140^ This dual functionality, combining UV attenuation and antimicrobial action, underscores the versatility and utility of ZnO NPs in safeguarding against the detrimental effects of UV radiation.^140^

In essence, the multifaceted approach underlying the UV protection conferred by ZnO NPs encompasses a synergy of optical properties, photostability, and antimicrobial effects. A comprehensive understanding of these mechanisms is pivotal for optimizing the design and application of ZnO NPs in a myriad of UV protection products. From advanced sunscreens and protective textiles to durable coatings and innovative biomedical devices, ZnO NPs offer a versatile and indispensable solution for shielding against the harmful effects of UV radiation in diverse settings.^141^

### 5.1 Exploration of the mechanisms by which ZnO NPs provide UV protection

Zinc oxide nanoparticles (ZnO NPs) offer UV protection through a series of intricate mechanisms that capitalize on their unique properties at the nanoscale. The mechanism underlying the UV protection provided by zinc oxide nanoparticles (ZnO NPs) primarily stems from their optical characteristics, prominently their ability to absorb and scatter ultraviolet (UV) radiation.^138^ This mechanism is intricately tied to the wide bandgap exhibited by ZnO material. The bandgap of a semiconductor like ZnO refers to the energy difference between the valence band and the conduction band, and in the case of ZnO, this bandgap is relatively wide, typically around 3.37 eV at room temperature.^142^

When exposed to UV radiation, ZnO NPs efficiently absorb photons within the UV spectrum due to their wide bandgap. This absorption process results in the promotion of valence band electrons to the conduction band, generating electron–hole pairs.^142,143^ These photoexcited charge carriers can subsequently participate in various processes such as photocatalysis or photovoltaic energy conversion.^143^ However, in the context of UV protection, the primary significance lies in the absorption of UV radiation, thereby preventing it from penetrating through the material.^143,144^

Furthermore, ZnO NPs also exhibit the phenomenon of scattering, wherein incident UV radiation interacts with the nanoparticles and is redirected in various directions.^145^ This scattering process is influenced by factors such as the size, shape, and surface morphology of the nanoparticles. As a result, a portion of the incident UV radiation is scattered away from the material, reducing the amount of radiation that can penetrate through and reach underlying materials or skin.^146^

The studies by Wu & Wu (2007),^147^ Ma *et al.* (2010),^148^ Pintupimol *et al.* (2023),^149^ Mishra *et al.* (2010),^150^ Indolia & Gaur (2012),^151^ and Vincent *et al.* (2018),^152^ collectively contribute to our understanding of this mechanism and its implications for UV protection.

Ma *et al.* (2010)^148^ provided insights into the optical properties of ZnO NPs, demonstrating their UV absorption capabilities through spectroscopic analyses. Wu & Wu (2007) discussed the importance of ZnO in photocatalysis and its potential for UV-visible photocatalytic applications.^147^ Pintupimol *et al.* (2023) explored different ZnO nanostructures and their optical properties, highlighting the superior UV photodetection capabilities of ZnO nanoparticle-based devices.^149^ Mishra *et al.* (2010) investigated the photoconductivity of ZnO NPs, shedding light on their UV-responsive behaviour.^150^ Indolia & Gaur (2012) studied the optical properties of ZnO nanoparticle-incorporated nanocomposite films, emphasizing their enhanced UV absorption.^151^ Finally, Vincent *et al.* (2018) explored the impact of ZnO nanoparticle size on optical properties and solar cell performance, indicating the potential for optimizing UV absorption and scattering effects.^152^

Overall, these studies collectively reinforce the importance of the optical characteristics of ZnO NPs, particularly their UV absorption and scattering capabilities, in providing effective UV protection. They underscore the versatility of ZnO NPs in various applications ranging from sunscreen formulations to photovoltaic devices, where their optical properties play a crucial role.

In addition to their UV absorption properties, ZnO NPs exhibit exceptional photostability, enabling them to sustain their UV-protective capabilities over prolonged exposure to sunlight or artificial UV sources.^153^ This sustained photostability is crucial for maintaining consistent UV protection, making ZnO NPs a reliable choice for applications requiring enduring UV shielding effects. For instance, the subsequent studies provided additional validation for the mechanisms mentioned above: Jun *et al.* (2009) embarked on a meticulous exploration of ZnO NP-based UV photodetectors, focusing on the nuanced interplay between photostability and optoelectronic performance.^154^ Their findings revealed a commendable degree of photostability exhibited by these devices, manifesting in sustained high on/off ratios even under prolonged UV irradiation. This exceptional endurance underscores the inherent stability of ZnO NPs, crucial for their continued functionality in demanding optoelectronic applications.

Shahabi-Ghahfarrokhi *et al.* (2015) took a nuanced approach to investigate the photostability of ZnO NPs integrated into kefiran biopolymers, offering insights into their role in bolstering mechanical and UV protective properties.^155^ Through meticulous analysis, they unveiled a synergistic enhancement in mechanical integrity and UV shielding efficacy in nanocomposites enriched with ZnO NPs. This augmentation underscores not only the photostability of ZnO NPs within the polymeric matrix but also their instrumental role in imparting durable UV protection to composite materials.

Ghamsari *et al.* (2016) delved into the intricacies of UV-protection conferred by sol–gel-derived thin ZnO films, probing their photostability over prolonged UV exposure periods.^156^ Their systematic investigation unraveled the enduring UV protection efficacy of these thin ZnO films, showcasing their remarkable photostability under challenging environmental conditions. Such findings underscore the intrinsic robustness of ZnO NPs within thin film configurations, affirming their resilience and long-term viability in UV protection applications.

El-hady *et al.* (2013) ventured into the realm of flame retardancy and UV protection, examining the integration of ZnO NPs into cellulosic fabrics.^157^ Through a comprehensive analysis, they unveiled a notable enhancement in UV protection without compromising mechanical integrity, attributable to the enduring photostability of ZnO NPs within the fabric matrix. This augmentation underscores the enduring stability and versatility of ZnO NPs, positioning them as indispensable assets in UV protective applications across various industries.

In comparing these studies, while Jun *et al.* (2009)^154^ and Shahabi-Ghahfarrokhi *et al.* (2015)^155^ primarily focus on immediate effects and mechanical properties, Ghamsari *et al.* (2016)^156^ and El-hady *et al.* (2013) explored the long-term photostability of ZnO NPs.^157^ They collectively contribute to our understanding of how ZnO NPs maintain their UV-protective capabilities over extended periods, providing valuable insights for various applications. In essence, these advanced studies collectively underscore the pivotal role of photostability in ensuring the sustained efficacy and reliability of ZnO NPs in UV protection applications. Their inherent resilience and ability to withstand prolonged UV exposure without compromising functionality or structural integrity exemplify their versatility and robustness, cementing their status as indispensable components in UV-protective materials and devices.

Furthermore, the antimicrobial attributes of ZnO NPs contribute to UV protection by inhibiting microbial growth on surfaces or skin exposed to UV radiation.^140^ By impeding the proliferation of microorganisms, ZnO NPs help uphold the integrity of the protective barrier, enhancing the overall efficacy of UV protection provided.^158,159^

Microorganisms are known to thrive in environments exposed to UV radiation, and their presence can compromise the effectiveness of UV protection measures. By inhibiting the growth of these microorganisms, ZnO NPs help maintain the integrity of the protective barrier, thus enhancing the overall efficacy of UV protection provided.^158,159^ The mechanism underlying this antimicrobial activity involves the interaction between ZnO NPs and microbial cells. ZnO NPs possess unique surface properties that enable them to interact with microbial membranes, disrupting their structure and function. This disruption leads to the inhibition of essential cellular processes, ultimately resulting in microbial growth inhibition or cell death.^158,159^

Furthermore, the photocatalytic properties of ZnO NPs also contribute to their antimicrobial activity.^160^ When exposed to UV radiation, ZnO NPs undergo photoexcitation, leading to the generation of reactive oxygen species (ROS) such as hydroxyl radicals and superoxide ions.^160^ These ROS exhibit strong oxidative potential and can induce oxidative stress in microbial cells, leading to damage to lipids, proteins, and DNA, thereby inhibiting microbial growth.^117^ Overall, the antimicrobial attributes of ZnO NPs complement their UV protection capabilities by effectively preventing microbial colonization and growth on surfaces or skin exposed to UV radiation. The multifaceted mechanisms underlying the UV protection conferred by ZnO NPs highlight the diverse ways in which these nanoparticles can safeguard against UV radiation. Understanding these mechanisms is critical for optimizing the design and application of ZnO NPs in a wide range of UV protection products, spanning from sunscreens and textiles to coatings and biomedical devices. Further research into the intricate interplay of these mechanisms can pave the way for enhanced UV protection strategies utilizing ZnO nanoparticles.

### 5.2 Review of recent advancements in utilizing ZnO NPs for UV-blocking applications in healthcare

In recent years, the exploration of nano-sized materials with exceptional properties has sparked significant interest, particularly in the realm of healthcare applications. Among these materials, zinc oxide nanoparticles (ZnO NPs) have emerged as promising candidates, owing to their remarkable flexibility, biocompatibility, and inherent ability to block ultraviolet (UV) radiation.^161^ The utilization of ZnO NPs in UV-blocking applications holds immense potential for various healthcare products, ranging from sunscreens to biomedical devices.^162,163^

A study by Cardozo *et al.* (2022) aimed to assess the efficacy of zinc oxide nanostructures of different sizes as UVA and UVB photoprotective components for sunscreen lotion.^164^ The interaction between light and nanoparticles was elucidated using Mie theory. The study quantified the UV protection provided by ZnO-based sunscreen formulations using the Sun Protection Factor (SPF), with optimal SPF values observed for nanoparticles ranging in diameter from 100 nm to 160 nm. Additionally, the study explored the impact of varying ZnO concentrations (20% w/w and 25% w/w) on SPF values. The rational use of ZnO nanospheres, coupled with the identification of optimized nanostructures, has the potential to reduce the quantity of nanostructured inputs required in the cosmetic manufacturing industry.

This research underscores the significant role of ZnO nanoparticles in enhancing the UV-blocking capabilities of sunscreen formulations. By leveraging the unique properties of ZnO NPs and optimizing their size and concentration, sunscreen products can achieve higher SPF values while minimizing the amount of nanostructured materials utilized. Such advancements not only contribute to the development of more effective and efficient sunscreens but also address concerns regarding the safe and sustainable use of nanostructured ingredients in cosmetic manufacturing. In another groundbreaking study by Rabani *et al.* (2021), the utilization of zinc oxide nanoparticles (ZnO NPs) for UV-blocking applications in healthcare was comprehensively investigated.^165^ Nano-sized materials with superior flexibility, biocompatibility, and UV-blocking properties have gained significant attention, especially those exhibiting photocatalytic activity.^165^ The study focuses on synthesizing a hybrid material consisting of ZnO NPs deposited on cellulose nanofibers (CNF) through an environmentally friendly sol–gel reaction, aiming to systematically explore the UV-blocking capabilities of the hybrid.

**Fig. 18:**
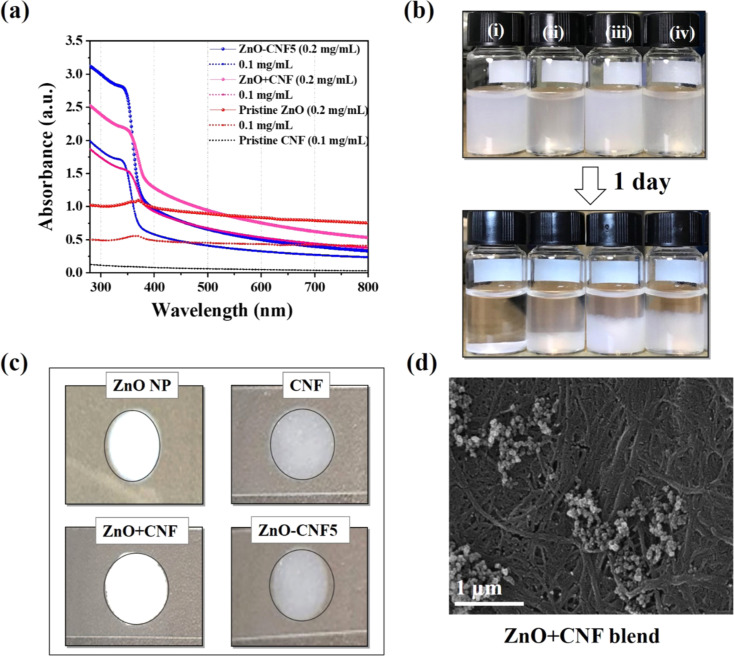
(a) UV-visible absorption spectroscopic analysis of ZnO–CNF5 hybrid, ZnO + CNF blend, and ZnO NP at 0.1 mg mL^−1^ and 0.2 mg mL^−1^ and pristine CNF sample at 0.1 mg mL^−1^, (b) dispersion stability at a high concentration such as (0.5 mg mL^−1^) (i) ZnO NP, (ii) pristine CNF, (iii) ZnO + CNF blend and (iv) ZnO–CNF5 hybrid, (c) the whitening effect drop-casted on the glass slide, (d) FE-SEM images of ZnO + CNF blend after dry.^165^

The researchers optimized the size of ZnO NPs on the CNF surface to enhance UV-blocking performance and transmittance. The resulting hybrid exhibited improved dispersion stability, minimal whitening effect, and high UV-blocking efficacy, with absorbance values of 3.05 a.u. and 2.80 a.u. in the UVB and UVA regions, respectively. The UV-visible (UV-vis) absorption spectra of ZnO–CNF hybrids concerning the ZnO NP size showcase variations in absorbance across different hybrid samples. The study highlights the inverse relationship between ZnO NP size and UV absorbance, emphasizing the importance of particle size control in optimizing UV-blocking properties. Additionally, the delicate balance between UV-blocking efficacy and visible light transparency is highlighted in Fig. 18, underscoring the significance of ZnO NP size and dispersion in attaining optimal UV protection while maintaining aesthetic appeal. The research reveals that smaller ZnO NPs and improved dispersion on the CNF surface led to increased UV absorbance and transparency in the visible region, crucial considerations for cosmetic applications.

**Fig. 19:**
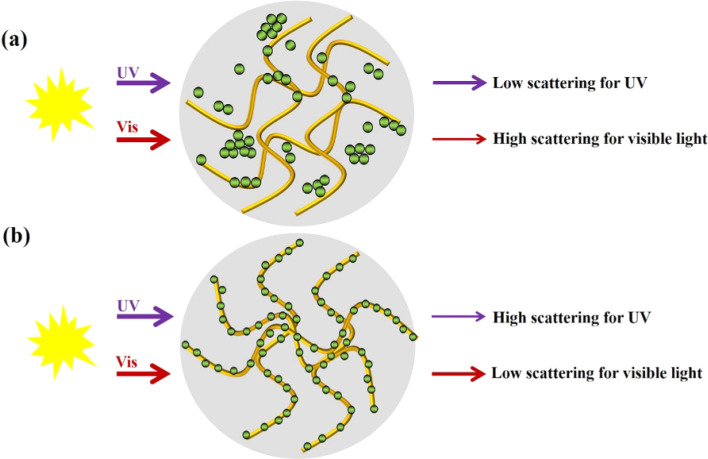
(a and b) Schematic drawing of the influence of ZnO particle size and incorporation on the CNF surface for the optical properties.^165^

The findings showcased in Fig. 19 illustrate how optimizing particle size and dispersion can significantly enhance UV-blocking effectiveness while maintaining visible light transparency. The study emphasizes the importance of dispersibility and particle size in bolstering UV-blocking properties, while also acknowledging the challenges of balancing UV protection with visible light transparency. In summary, the study conducted by Rabani *et al.* (2021) offers significant insights into the utilization of ZnO NPs for UV-blocking applications in healthcare.^165^ Through meticulous control of particle size and improved dispersion, the developed ZnO–CNF hybrids present a promising avenue for the creation of advanced UV-blocking products. These hybrids not only demonstrate enhanced efficacy but also possess aesthetic appeal, marking a substantial advancement in UV protection technology.

A study by Huang *et al.* (2020) highlighted the application of ZnO NPs in addressing polymorphous light eruption (PLE), an acquired idiopathic photodermatosis primarily induced by excessive UV radiation exposure.^142^ The study focused on the synthesis of acetyl-11-keto-β-boswellic acid (AKBA) loaded ZnO nanoparticles, wherein the drug release behavior was controlled by UV radiation. These nanoparticles not only exhibited UV-reflective properties but also efficiently released AKBA upon UV exposure, which possesses excellent antioxidant and anti-inflammatory effects. Moreover, the biocompatibility of these nanoparticles with HaCaT cells further enhances their potential for PLE protection and therapy.

AKBA@ZnO NPs offer a dual functionality of UV protection and cytoprotection simultaneously, making them a promising candidate for addressing various skin conditions. The therapeutic effects of AKBA are attributed to its ability to reduce inflammatory factors and activate cytoprotective pathways, thereby safeguarding cells under stress. Furthermore, AKBA demonstrates potential anticancer properties by inhibiting cell proliferation and inducing apoptosis in various cancer cell lines.

The synergistic combination of ZnO nanoparticles and AKBA holds great promise in anti-cancer therapies, with a focus on activating cytoprotective pathways while inhibiting apoptotic mechanisms. ZnO nanoparticles, known for their superior drug delivery capabilities, are utilized in this context not only to deliver AKBA but also to mitigate the harmful effects of UV radiation by reflecting and absorbing photons.

In a nutshell, AKBA-loaded ZnO nanoparticles represent a novel approach for UV protection and medicine administration in PLE protection and therapy. With their UV-controlled drug release behaviour, excellent biocompatibility, and multifunctional properties, these nanoparticles offer a versatile solution for addressing various skin conditions and hold promise for further advancements in healthcare applications.

Lin *et al.* (2019) conducted a comprehensive investigation into the role of ZnO NPs in protecting against UV-induced photodamage during wound healing.^166^ Their study stands out for its focus on the cellular level responses to UV radiation, particularly in the context of wound healing. The findings regarding the enhancement of cell survival with the addition of spherical ZnO NPs are intriguing, suggesting a promising avenue for utilizing these nanoparticles in skincare formulations targeting UV protection during the wound healing process. Furthermore, the observed reduction in p53 expression highlights the potential of ZnO-containing barrier films in minimizing UV-induced cellular damage, which could have significant implications for improving wound healing outcomes.

On the other hand, the study by Mohammed *et al.* (2019) is pivotal in evaluating the potential of zinc oxide nanoparticles (ZnO NPs) as a UV protection agent, especially considering their repeated application on human skin.^167^ The safety assessment conducted in this research is critical, as it addresses a fundamental concern in the development of skincare products that incorporate nanoparticles. Using advanced imaging techniques, the study meticulously visualized ZnO-NP penetration and assessed skin toxicity, providing a comprehensive understanding of the interaction between ZnO NPs and human skin.

The findings of this study are highly reassuring for the potential use of ZnO NPs in UV protection products. The results demonstrated no evidence of ZnO-NP penetration into the viable epidermis, which means that the nanoparticles do not reach the deeper layers of the skin. This is a crucial aspect of safety, as deeper penetration could potentially lead to systemic exposure and adverse health effects. By confirming that ZnO NPs remain confined to the outermost layers of the skin, the study alleviates major concerns regarding their use in topical applications. Additionally, the study found no signs of local toxicity, further supporting the safety of ZnO NPs when used repeatedly on the skin. This absence of local toxicity is vital for products intended for daily use, such as sunscreens and other UV protection skincare items. The lack of adverse skin reactions indicates that ZnO NPs can be incorporated into formulations without causing irritation or damage to the skin, making them suitable for long-term use.

These safety assurances are significant because they complement the well-documented UV-blocking properties of ZnO NPs. Zinc oxide is known for its ability to effectively absorb and scatter UV radiation, thereby protecting the skin from harmful UV rays. The study by Mohammed *et al.*^167^ (2019) enhances the confidence in using ZnO NPs as a UV protection agent by providing robust evidence that they are safe for human use, even with repeated application.

In contrast, Ghamsari *et al.* (2016) focused on the development of sol–gel-derived thin ZnO films for UV protection.^156^ Their study offers valuable insights into the engineering of UV-blocking coatings using ZnO nanoparticles. The demonstration of high UV-protection factor and tunable optical properties of the thin ZnO films underscores their potential for commercial applications in skincare and other industries requiring UV protection. Additionally, the optimization of sol and dopant ion concentrations highlights the importance of material engineering in enhancing UV-blocking efficacy.

Comparing these studies, each contributes unique perspectives to the utilization of ZnO NPs for UV protection. Lin *et al.* (2019) shed light on the cellular mechanisms underlying the photoprotective effects of ZnO NPs during wound healing,^166^ while Mohammed *et al.* (2019) addressed safety concerns associated with their repeated application on human skin.^167^ Ghamsari *et al.* (2016) focused on the development of engineered coatings for UV protection, highlighting the potential for practical applications.^156^ Collectively, these studies underscore the multifaceted nature of ZnO NPs in UV protection and highlight the need for further research to optimize their efficacy and safety in skincare formulations.

Two seminal studies, conducted by Ghamsari *et al.* (2016)^156^ and Huang *et al.* (2013),^142^ epitomize this trend, each offering unique insights into the utilization of ZnO NPs for UV protection albeit through different approaches. While Ghamsari *et al.* (2016) delve into the development of UV-blocking coatings using sol–gel-derived thin ZnO films,^156^ Huang *et al.* (2013) pioneer an intelligent drug delivery system leveraging the capabilities of ZnO NPs for skincare.^142^ Despite their distinct methodologies, both studies underscore the potential of ZnO NPs in addressing critical challenges in skincare and healthcare, paving the way for innovative solutions in UV protection and beyond. Ghamsari *et al.* (2016)^156^ embarked on an extensive exploration of the UV-protection capabilities inherent in sol–gel-derived thin ZnO films, presenting a study that offers profound implications for skincare and related industries.^152^ Their investigation commenced with an assessment of the optical properties of the prepared ZnO films, revealing a remarkable transparency in the visible wavelength region coupled with an effective blocking of UV radiation. The observed UV-protection factor of 50+ underscores the potential of these films as robust barriers against harmful UV rays, crucial for safeguarding the skin from photodamage.

Furthermore, the study investigated the optimization of sol and dopant ion concentrations, shedding light on the critical parameters influencing the UV-blocking efficacy of the thin ZnO films. By systematically evaluating the impact of these factors, the researchers emphasized the importance of material engineering in enhancing UV protection, highlighting opportunities for further refinement in the design of protective coatings.

Additionally, the incorporation of impurities such as indium zinc oxide (IZO) was identified as a strategy to augment UV protection, opening avenues for novel approaches in enhancing the photoprotective properties of ZnO-based materials. Through meticulous characterization of the optical properties of both thin ZnO and IZO films, the study showcased their effectiveness in shielding UV radiation, reaffirming their potential for diverse applications in skincare and artificial skin industries.

In contrast, Huang *et al.* (2013) presented a pioneering investigation into a UV and dark-triggered drug delivery system harnessing the capabilities of zinc oxide nanoparticles (ZnO NPs).^142^ Their study heralds a paradigm shift in drug delivery technology, introducing an intelligent system designed to release a UV-absorption medicine, benzophenone-3 (Bp-3), upon UV exposure and re-encapsulate it in the dark.

The research commenced with the synthesis and characterization of ZnO NPs, elucidating their dynamic properties under UV and dark exposure. Leveraging the intrinsic photosensitive properties of ZnO NPs, the study demonstrated their versatility as carriers for Bp-3, optimizing encapsulation efficiency and loading capacity to facilitate controlled drug release.

Significantly, the study unveiled the remarkable release kinetics of Bp-3 from ZnO NPs, showcasing their ability for repetitive on-demand drug delivery triggered by UV exposure and subsequent re-encapsulation in the dark. This intelligent behaviour highlights the potential of ZnO NPs as multifunctional agents, capable of serving as both drug carriers and UV filters, thereby offering a synergistic approach to skin protection. Moreover, cytotoxicity studies confirmed the biocompatibility of Bp-3 loaded ZnO NPs, underscoring their suitability for skincare applications. While the study presents promising results, it acknowledges the need for further research to comprehensively evaluate the long-term effects and safety profile of Bp-3 loaded ZnO NPs, emphasizing the importance of rigorous testing before clinical implementation.

In summary, both studies contribute valuable insights into the utilization of zinc oxide nanoparticles for UV protection, albeit through different approaches. While Ghamsari *et al.* (2016)^156^ focused on the development of UV-blocking coatings,^152^ Huang *et al.* (2013) explored the innovative application of ZnO NPs in drug delivery systems for skincare.^142^ Together, these studies showcase the versatility and potential of ZnO NPs in addressing diverse challenges in healthcare, paving the way for future advancements in skin protection and drug delivery technology.

In a very recent study, Elbrolesy *et al.* (2023) conducted a study aiming to synthesize multifunctional zinc oxide nanoparticles (ZnO NPs) using *Solanum lycopersicum* (SL) fruit juice for applications as antibacterial, anticancer, and UV sunscreen agents.^168^ The synthesized ZnO NPs were characterized for their optical properties, cytotoxicity against human lung fibroblast (WI-38) and hepatocellular carcinoma (HePG2) cell lines, and antibacterial activity against *Escherichia coli* and *Staphylococcus aureus*. The antioxidant activity and *in vitro* sun protection factor (SPF) of the nanoparticles were also evaluated.

The results revealed that the ZnO NPs exhibited a pure phase structure and characteristic functional groups, as confirmed by XRD, FTIR, and UV-Vis diffuse reflectance analysis. SEM images depicted quasi-spherical ZnO NPs with an average size of approximately 39 ± 12 nm. Remarkably, the ZnO NPs demonstrated high potency as sunscreens, displaying an *in vitro* SPF value of 16.8. The UV-visible diffused reflectance spectrum showed low reflectance values across the UV spectrum, indicating efficient absorption of ultraviolet rays by the ZnO NPs. The band gap energy of the ZnO NPs was calculated to be 3.16 eV, confirming their ability to absorb ultraviolet radiation effectively.

Furthermore, the study highlighted the triple role of SL fruit juice in the synthesis process, acting as a solvent, reducing agent, and stabilizer, which facilitated the production of ZnO NPs with antibacterial and anticarcinogenic properties. The synthesized ZnO NPs showed enhanced cytotoxic activity against hepatocellular carcinoma cells and exhibited antibacterial activity against pathogenic bacteria.

**Fig. 20:**
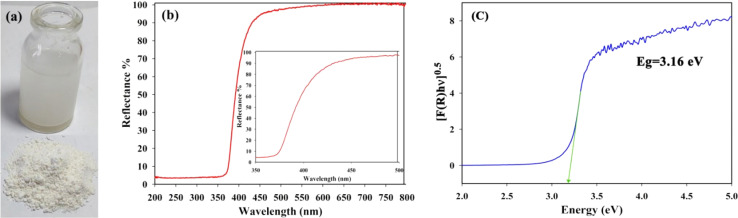
Optical properties of ZnO NPs: (a) white color of ZnO NPs, (b) UV-visible diffuse reflectance spectra of ZnO NPs (inset: zoomed in spectra in the range from 350 to 500 nm), and (c) plot of [*F*(*R*)*h*_]0.5 against photon energy (*hν*).^168^

Fig. 20 illustrates the optical properties and sun protection factor (SPF) performance of the synthesized ZnO NPs. The UV-visible diffused reflectance spectrum showed very low reflectance values in the entire UV region, indicating efficient absorption of ultraviolet rays by the ZnO NPs. The band gap energy of the ZnO NPs was calculated to be 3.16 eV, confirming their capability to absorb ultraviolet radiation effectively. The *in vitro* SPF value of the ZnO NPs was determined to be 16.8, suggesting their potential effectiveness as sunscreens in protecting the skin from the harmful effects of UV rays, in accordance with FDA guidelines for sunscreens.

Overall, the findings suggest that ZnO NPs synthesized using SL juice hold promise for further development in sunscreen formulations and personal care products, offering effective protection against the harmful effects of UV radiation.

The utilization of zinc oxide nanoparticles (ZnO NPs) in various applications has garnered significant attention owing to their exceptional properties, particularly in UV protection, antimicrobial activity, and material reinforcement. In the quest for advanced sunscreen formulations, Jo *et al.* (2019) directed their focus towards enhancing the UV-protective capabilities of ZnO by applying coatings of chitosan or niacinamide.^169^ In the study, transmission measurements were conducted by dispersing 0.001% of each ZnO, CS/ZnO, and Nia/ZnO sample in distilled water. The results revealed that the transmission of the synthesized materials, CS/ZnO and Nia/ZnO, was lower than that of pure ZnO, especially in the UV ray range (200–400 nm). The transmission difference between pure ZnO and CS/ZnO was 34.7%, while the difference between pure ZnO and Nia/ZnO was 39%. However, the reflectance differences between the materials were not significant in the visible ray region, although CS/ZnO and Nia/ZnO exhibited lower reflectance compared to ZnO in the UV ray region.

Moreover, the study evaluated the Sun Protection Factor (SPF) of sunscreen emulsions formulated with ZnO, CS/ZnO, and Nia/ZnO. The SPF measurements, conducted at 0 min and 15 min, demonstrated that the CS/ZnO and Nia/ZnO emulsions provided remarkable UV ray protection efficiency compared to the ZnO emulsion. However, CS/ZnO was found ineffective as a sunscreen emulsion due to its significantly higher UV ray transmission, transmitting more than 90% of UV rays. Additionally, the SPF mean for CS/ZnO was the lowest among the three materials, measuring only 1.18 at 15 min. Conversely, Nia/ZnO exhibited an SPF mean of 9.96 at 15 min and demonstrated a UV ray transmission of 16%, indicating its superior efficiency compared to pure ZnO.

**Fig. 21:**
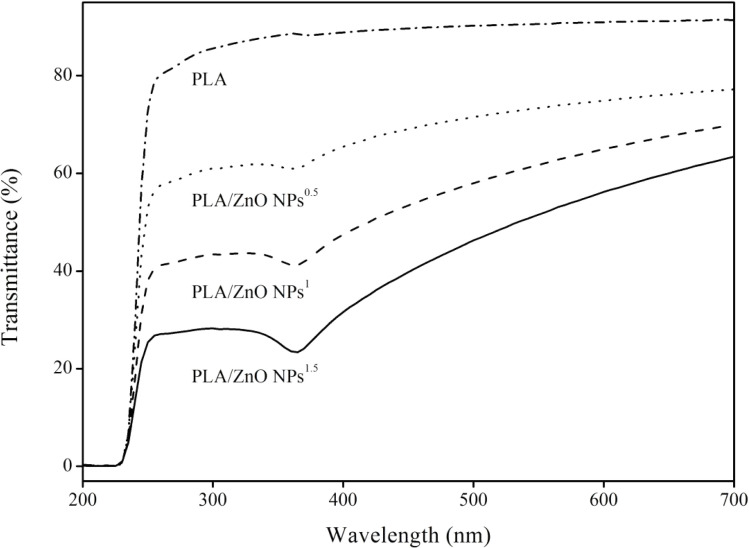
Light transmittance spectra of PLA and PLA/ZnO NPs composite films.^161^

In a parallel effort, Shankar *et al.* (2018) embarked on a problem-solving initiative aimed at addressing multifaceted challenges in food packaging through the integration of zinc oxide nanoparticles (ZnO NPs) into polylactic acid (PLA) composite films.^161^ The primary objectives were to enhance UV protection and antimicrobial activity, critical requirements for ensuring food safety and preservation in packaging materials. Their study involved evaluating the light transmittance of the composite films across a range of wavelengths (200–700 nm) to assess their optical properties (Fig. 21). Initially, the neat PLA film exhibited high transparency against both UV and visible light, indicated by significant light transmittance between 250-700 nm. However, upon the incorporation of ZnO NPs, a notable decrease in light transmittance was observed. This decrease was directly proportional to the ZnO NP content, with higher concentrations leading to more substantial reductions in transmittance.

The decline in light transmittance was attributed to the light impenetrable nature of ZnO NPs dispersed within the polymer matrix, preventing the passage of light. Interestingly, the decrease in transmittance was more pronounced in the UV light range compared to the visible light range. This finding indicated that the composite films effectively prevented UV penetration while slightly compromising overall transparency. The strong UV-light absorption property of ZnO NPs contributed significantly to the decrease in transmittance within the UV light range. This effect aligns with previous studies demonstrating the UV screening capabilities of ZnO NPs in various biopolymer-based films, including gelatin, agar, carrageenan, and carboxymethyl cellulose.

The PLA/ZnO NPs composite films exhibited notable UV screening capacity, particularly against UV-B (280–320 nm) and UV-A (320–400 nm) wavelengths. This property renders them suitable for applications in UV-light prevention food packaging, effectively preventing photocatalytic reactions in packaged foods and preserving their quality and safety.

**Fig. 22:**
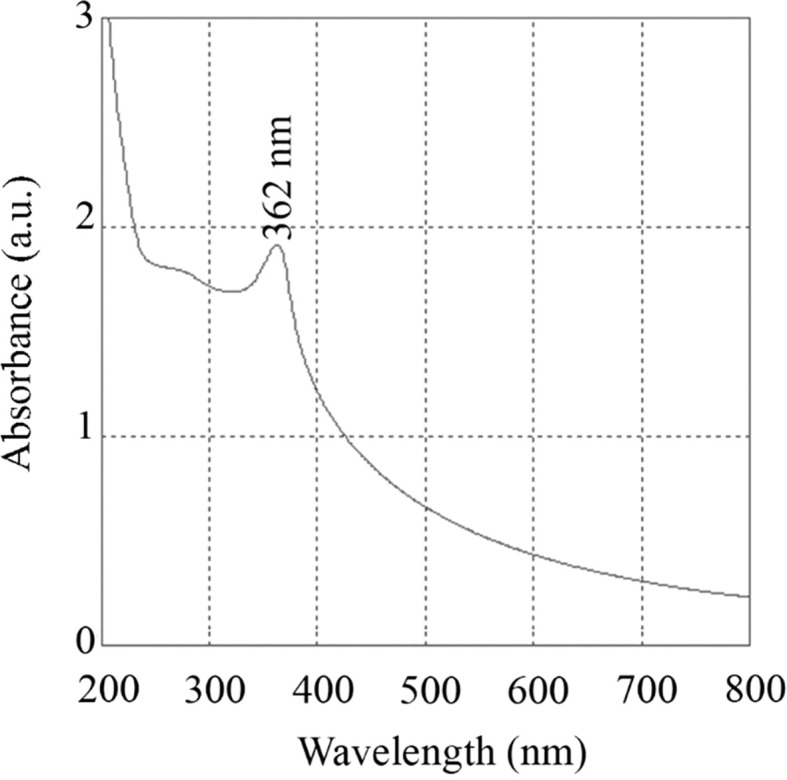
UV-Vis spectrum of the prepared ZnO nanorods, exhibiting an absorption peak at 362 nm.^162^

Additionally, Taghizadeh *et al.* (2020) investigated the UV-blocking properties of zinc oxide nanorods.^162^ The investigation focused on the potential of the prepared ZnO nanorods to absorb UV-Vis irradiation, with the spectrum depicted in Fig. 22. Notably, the nanorods exhibited an absorption peak at 362 nm, characteristic of ZnO nanostructures' absorption behaviour.

This unique property underscores the suitability of ZnO particles for producing valuable pharmaceutical compounds such as sunscreens, as they serve as efficient sun blockers offering protection against the adverse effects of UV light, particularly against UVA radiation. It's worth mentioning that combining ZnO with titanium dioxide (TiO_2_) particles is a common strategy to achieve broadband UV protection. Moreover, the presence of a shoulder in the UV-Vis spectrum indicates the efficacy of the prepared nanorods against both UVB (290–320 nm) and UVA (320–400 nm) radiations. The influence of UV illumination further enhances the biological activity of ZnO nanostructures. ZnO demonstrates high photocatalytic efficiency, and its ability to absorb UV light significantly enhances its interaction with bacterial cells, thereby facilitating growth inhibition or cell death through the generation of reactive oxygen species (ROS).

Moreover, Clar *et al.* (2019) investigated the environmental implications of ZnO NPs in surface coatings, shedding light on their release dynamics under environmental weathering conditions.^170^ The research explored the effectiveness of ZnO NPs in safeguarding wood surfaces from UV-induced degradation by examining the release of zinc over time from both pre-weathered and pristine boards. Initially, the study reveals that the highest release of zinc occurs during the first few sampling events, with water-based applications releasing significantly more zinc compared to stain-based applications. This indicates that ZnO NPs form a robust protective layer that is particularly effective at the onset of application. As the wood is exposed to environmental conditions over time, the zinc release rate stabilizes, suggesting that ZnO NPs remain adhered to the surface, continuing to provide UV protection.

Pre-weathering, simulating natural aging and environmental exposure, plays a crucial role in the behaviour of ZnO NPs. Pre-weathered boards, subjected to UV light and other weathering factors, exhibit initial moisture loss, which enhances the penetration of ZnO NPs into the wood. This deeper penetration results in lower initial zinc release from pre-weathered boards compared to pristine ones, as the nanoparticles are less susceptible to being washed away or removed through dermal contact. Despite these initial differences, the long-term release patterns for both pre-weathered and pristine boards converge, indicating that the UV protection provided by ZnO NPs is durable and stable over extended periods. The study emphasizes that the choice of dispersion medium significantly affects the performance of ZnO NPs, with stain-based applications demonstrating more sustained retention and, consequently, longer-lasting UV protection.

Overall, the findings highlight the efficacy of ZnO NPs in providing durable UV protection for outdoor wood applications. The nanoparticles' ability to absorb and scatter UV radiation effectively protects the wood from structural and aesthetic damage caused by prolonged UV exposure. The environmental stability of ZnO NPs, demonstrated by their consistent performance under varying conditions, underscores their suitability for enhancing the lifespan and performance of pressure-treated lumber. This study by Clar *et al.* (2019)^170^ thus underscores the importance of ZnO NPs as a protective agent in wood treatments, offering both immediate and long-term benefits in UV protection.

Lastly, the study by Homthawornchoo *et al.* (2022) explored the UV protection properties of zinc oxide nanoparticles (ZnO NPs) when incorporated into rice starch–gelatin (RS–G) composite films.^171^ This work highlighted the significant impact of ZnO NPs on the optical properties of these nanocomposite films, particularly their ability to block UV and visible light. The primary focus is on how varying concentrations of ZnO NPs affect light transmission and transparency, critical factors for applications requiring UV protection.^172^

The study shows that the addition of ZnO NPs to RS–G composite films drastically reduced their UV-light transmission. The control RS–G films, which do not contain ZnO NPs, exhibit considerable UV-light transmission, ranging from 0.06% to 67.80% at wavelengths between 200 and 280 nm. In contrast, films with higher concentrations of ZnO NPs (>0.5%, w/v) completely block UV light, indicating no transmission. This demonstrates the strong UV-shielding capability of ZnO NPs, making the composite films highly effective as UV barriers.^173^

In addition to UV light, the presence of ZnO NPs also significantly reduces the transmission of visible light (400–800 nm). The control RS–G films allow 75.07% to 83.15% of visible light to pass through, whereas the RS–G–ZnONPs nanocomposite films show a markedly lower transmission range of 0.33% to 18.82%. This reduction in visible light transmission is attributed to light scattering caused by the ZnO NPs within the film matrix. Such light scattering is a common phenomenon in nanocomposite films and contributes to their overall opacity.^174^

The transparency measurements further illustrate the impact of ZnO NPs on the RS–G films. The control films without ZnO NPs exhibited the highest transparency. However, the inclusion of ZnO NPs significantly decreases the films' transparency, with the most opaque film being the RS–G composite with 3% ZnO NPs (w/v). This reduced transparency is linked to the addition of ZnO NPs and their compatibility with the biopolymer matrix. While such opacity might render the films unsuitable for see-through packaging applications where product visibility is essential, it makes them excellent candidates for UV-light barrier packaging. This is particularly beneficial for packaging fatty foods prone to oxidative deterioration, as effective UV shielding can extend shelf life and preserve product quality.^175^

In general, Homthawornchoo *et al.* (2022)^171^ demonstrate that ZnO NPs significantly enhance the UV protection properties of RS–G composite films by effectively blocking UV and visible light transmission and increasing film opacity. These findings suggest that ZnO NP-incorporated films have substantial potential as UV-light barrier packaging materials, particularly for applications where preventing oxidative damage is crucial. This study underscores the versatility and effectiveness of ZnO NPs in developing advanced materials for UV protection.^176^

Collectively, these studies highlight the problem-solving perspective in addressing various challenges and opportunities associated with ZnO NPs across different domains.^177^ By leveraging advanced synthesis methods and sustainable approaches, researchers have developed tailored solutions to meet specific application requirements, ranging from UV protection and antimicrobial activity to environmental sustainability and material reinforcement.^178^

### 5.3 Factors affecting the UV protective performance of ZnO NPs

Zinc oxide nanoparticles (ZnO NPs) are widely recognized for their ability to provide effective UV protection, making them valuable in various industries such as textiles, cosmetics, and coatings.^179^ However, the efficacy of ZnO NPs in UV protection is influenced by several key factors: particle size, dispersion, and coating.^180^

The size of ZnO nanoparticles is critical in determining their UV protective performance.^181–184^ Smaller nanoparticles have a larger surface area-to-volume ratio, allowing for more efficient light scattering and absorption.^181,182^ This results in enhanced UV protection compared to larger particles, which scatter and reflect UV radiation less effectively. Therefore, optimizing the particle size of ZnO NPs is essential for maximizing their UV blocking capabilities.^181–184^

The dispersion of ZnO nanoparticles within a medium or matrix is another important factor affecting their UV protective properties. Well-dispersed nanoparticles ensure uniform coverage and interaction with incident UV radiation, leading to more effective attenuation of harmful UV rays.^155,156^ Conversely, poor dispersion can result in agglomeration or clustering of nanoparticles, compromising the overall UV protection. Thus, meticulous attention to dispersion techniques is crucial for harnessing the full UV protective potential of ZnO NPs.^157^

Coating ZnO nanoparticles with suitable materials or additives can further enhance their UV blocking efficiency and stability.^185,186^ Functional coatings improve the compatibility of ZnO NPs with various substrates, promote uniform dispersion, and provide additional UV absorption or reflection properties. Moreover, coatings may offer benefits such as enhanced durability, water resistance, and photostability, thereby prolonging the lifespan and effectiveness of UV protective materials incorporating ZnO NPs.^186^ Careful selection and optimization of coating materials are essential for maximizing the UV blocking performance and overall functionality of ZnO-based UV protective formulations.^185,186^

In a nutshell, the UV protective performance of ZnO nanoparticles is influenced by factors such as particle size, dispersion, and coating. Understanding the complex interplay of these factors is crucial for optimizing the design and performance of ZnO-based UV protective materials across various applications. By addressing these factors, researchers can continue to innovate and develop ZnO nanoparticle-based solutions with enhanced UV blocking efficacy, durability, and versatility.

## 6 Challenges and future perspectives

The integration of zinc oxide nanoparticles (ZnO NPs) for antimicrobial and UV protective healthcare solutions presents several challenges that need to be addressed for optimal performance and efficacy. One of the key challenges is the identification of suitable methods for incorporating ZnO NPs into healthcare products while ensuring their stability, compatibility, and safety for use in medical settings.^161^ Additionally, achieving a balance between antimicrobial activity and biocompatibility is essential to prevent adverse effects on human health.^187^

To overcome these challenges, researchers and developers are exploring various strategies. One approach involves optimizing the synthesis and surface modification of ZnO NPs to enhance their antimicrobial efficacy while minimizing cytotoxicity. This may include tailoring the size, shape, and surface chemistry of ZnO NPs to improve their interactions with microbial pathogens and reduce their impact on mammalian cells.^188^

Furthermore, the development of nanocomposite materials offers a promising avenue for enhancing the performance of ZnO-based healthcare solutions.^189^ By incorporating ZnO NPs into biocompatible polymers or other matrices, researchers can create multifunctional materials with synergistic antimicrobial and UV protective properties.^190^ These nanocomposites can be tailored to specific applications, such as wound dressings, medical textiles, or surface coatings for medical devices.

Surface modification techniques also play a crucial role in overcoming challenges associated with the integration of ZnO NPs into healthcare products.^191^ Functionalizing the surface of ZnO NPs with biomolecules or polymers can improve their stability, biocompatibility, and targeted delivery to infection sites. Additionally, surface coatings can be designed to enhance the adhesion of ZnO NPs to various substrates, ensuring uniform coverage and long-term durability.^191^

Looking ahead, future directions in this field may involve the development of advanced nanocomposite materials with enhanced functionalities, such as controlled drug release or stimuli-responsive behaviour.^192,193^ Furthermore, emerging trends in surface modification techniques, such as plasma treatment or biomimetic coatings, hold promise for improving the performance and versatility of ZnO-based healthcare solutions.^193,194^

In summary, while challenges exist in the integration of ZnO NPs for antimicrobial and UV protective healthcare solutions, innovative strategies and emerging trends offer opportunities for overcoming these obstacles. By addressing these challenges and exploring future directions, researchers can contribute to the development of safer, more effective, and versatile healthcare products that harness the unique properties of ZnO NPs.

## 7 Conclusions

The exploration of zinc oxide nanoparticles (ZnO NPs) has unveiled a rich tapestry of insights into their pivotal role in advancing healthcare solutions, particularly in combating microbial infections and providing effective UV protection. From the foundational studies elucidating the optical characteristics of ZnO NPs to the in-depth investigations into their photostability over extended UV exposure durations, the journey has illuminated the multifaceted nature of these nanostructures. The synthesis and characterization endeavors undertaken in various studies have provided invaluable contributions to understanding ZnO NPs' mechanisms and properties. These investigations, spanning from immediate effects to long-term stability assessments, have not only elucidated the immediate effects and mechanical properties but have also shed light on the enduring photostability of ZnO NPs, essential for sustained UV protection. Moreover, the exploration has extended to the integration of ZnO NPs into healthcare products through innovative approaches like nanocomposite materials and surface modification techniques. These strategies have been instrumental in harnessing the antimicrobial attributes of ZnO NPs, effectively inhibiting microbial growth and bolstering the overall efficacy of UV protection. In essence, the findings underscore the imperative of incorporating ZnO NPs into advanced healthcare solutions, leveraging their unique properties to address microbial infections and provide robust UV protection. Nonetheless, the journey toward fully harnessing the potential of ZnO NPs in healthcare applications requires continued research endeavours and collaborative efforts. By persisting in exploration and innovation, a path can be forged toward the development of safer, more effective, and versatile healthcare products, benefiting human health on a global scale.

## Data availability

The study, “Cutting-edge developments in zinc oxide nanoparticles: synthesis and applications for enhanced antimicrobial and UV protection in healthcare solutions,” is a review article and does not involve the generation of original experimental data. The information presented is derived from previously published studies and existing literature. All relevant sources and references are cited within the manuscript. For access to the original data and studies reviewed in this article, readers are encouraged to consult the cited references directly. Permissions have been obtained to use all previously published figures and data included in this review.

## Author contributions

The manuscript was drafted and revised by all the authors.

## Conflicts of interest

On behalf of all authors, the corresponding author states that there is no conflict of interest.
