# Correction: Cutting-edge developments in zinc oxide nanoparticles: synthesis and applications for enhanced antimicrobial and UV protection in healthcare solutions

**DOI:** 10.1039/d5ra90105g

**Published:** 2025-09-30

**Authors:** Egwonor Loveth Irede, Raymond Femi Awoyemi, Babatunde Owolabi, Omowunmi Rebecca Aworinde, Rofiat Odunayo Kajola, Ajibola Hazeez, Ayuba Adawale Raji, Latifat Oluwatobi Ganiyu, Chimezie O. Onukwuli, Asishana Paul Onivefu, Ikhazuagbe Hilary Ifijen

**Affiliations:** a Department of Chemistry, Clemson University Clemson South Carolina USA; b Department of Chemistry, Mississippi State University Starkville Mississippi MS 39762 USA; c Department of Civil Engineering, University of Alabama Tuscaloosa Alabama AL 35487 USA; d Department of Chemistry, Michigan Technological University Houghton Michigan USA; e Department of Biomedical Engineering, University of Rochester 500 Joseph C. Wilson Blvd. Rochester NY 14627 USA; f Department of Urban and Regional Planning, University of Lagos Lagos Nigeria; g Department of Surveying and Geo-Informatics, Bells University of Technology Ota Ogun State Nigeria; h Department of Chemistry, Brigham Young University Provo Utah USA; i Department of Chemistry, Eastern New Mexico University Portales New Mexico USA; j University of Benin Benin City Edo State Nigeria; k Department of Research Outreach, Rubber Research Institute of Nigeria Iyanomo Benin City Nigeria larylans4u@yahoo.com ifijen.hilary@rrin.gov.ng

## Abstract

Correction for ‘Cutting-edge developments in zinc oxide nanoparticles: synthesis and applications for enhanced antimicrobial and UV protection in healthcare solutions’ by Egwonor Loveth Irede *et al.*, *RSC Adv.*, 2024, **14**, 20992–21034, https://doi.org/10.1039/D4RA02452D.

The authors regret that the affiliation for Asishana Paul Onivefu (affiliation *j*) was incorrectly shown in the original manuscript. The corrected list of affiliations is as shown here.

The Royal Society of Chemistry apologises for these errors and any consequent inconvenience to authors and readers.

